# Cell-intrinsic platinum response and associated genetic and gene expression signatures in ovarian cancer

**DOI:** 10.1038/s41417-025-00941-5

**Published:** 2025-07-19

**Authors:** Kristin M. Adams, Jae-Rim Wendt, Josie Wood, Sydney Olson, Ryan Moreno, Zhongmou Jin, Srihari Gopalan, Jessica D. Lang

**Affiliations:** 1 Center for Precision Medicine, University of Wisconsin-Madison, Madison, WI USA; 2 Department of Computer Science, University of Wisconsin-Madison, Madison, WI USA; 3 Department of Pathology and Laboratory Medicine, University of Wisconsin-Madison, Madison, WI USA

**Keywords:** Ovarian cancer, Cancer genetics, Gene expression, Ovarian cancer, Cancer genomics

## Abstract

Ovarian cancers are still largely treated with platinum-based chemotherapy as the standard of care, yet few biomarkers of clinical response have had an impact on clinical decision making. Previous work has relied on poor models of the most common subtypes of epithelial ovarian cancers and necessitates a careful examination of the most suitable in vitro models. We performed extensive drug dose response assays and gene expression profiling on 36 ovarian cancer cell lines across over seven subtypes. This is the largest quantitative database of quantitative cisplatin and carboplatin response in ovarian cancer cell lines. Our results demonstrate that cell lines largely fall either well above or below the clinical maximally achievable dose (C_max_) of each compound. We performed differential expression analysis for high-grade serous ovarian carcinoma cell lines. Further, we generated two platinum-resistant derivatives each for OVCAR3 and OVCAR4. Combined with clinically resistant PEO1/PEO4/PEO6 and PEA1/PEA2 isogenic models, we performed differential expression analysis for seven platinum-resistant isogenic pairs. Common themes in differential expression were innate immunity/STAT activation, epithelial-to-mesenchymal transition (EMT) and stemness, and platinum influx/efflux regulators. We also performed copy number signature analysis and orthogonal measures of homologous recombination deficiency (HRD) scar scores and copy number burden, which is the first report to our knowledge applying field-standard copy number signatures to ovarian cancer cell lines. We also examined markers and functional readouts of stemness that revealed that cell lines are poor models for examination of stemness contributions to platinum resistance, suggesting that this is a transient state. Overall, this study serves as a resource to determine the best cell lines to utilize for ovarian cancer research on certain subtypes and platinum response studies, as well as sparks new hypotheses for future study in ovarian cancer.

## Introduction

Ovarian cancers (OVCA), of which high-grade serous ovarian carcinoma (HGSOC) is the most common subtype, account for 5% of all cancer deaths in females in the United States [1]. OVCA patients receive platinum-based chemotherapy (carboplatin, cisplatin) [2], but over half of patients are diagnosed at Stage IV and face a dismal 31.5% five-year survival rate [3]. Overall, the progression free interval for first-line OVCA is ~18 months, and around 80% of patients are considered sensitive to platinum-based chemotherapy [4]. However, patients initially sensitive to platinum-based chemotherapy display decreased progression-free intervals and response rates on platinum with each subsequent recurrence [5]. Currently, the only way to determine if a patient is responsive to platinum-based chemotherapy is to treat them and wait up to six months to determine response.

Platinum-based chemotherapies bind to DNA to form inter- and intrastrand DNA adducts that block replication, leading to cell cycle arrest and induction of apoptosis [6]. These adducts are repaired by a variety of DNA damage repair (DDR) mechanisms, which can become altered as a mechanism of resistance [7–9]. Many other distinct mechanisms of resistance to platinum have been described, involving over 900 implicated genes to date, which are reviewed elsewhere [10]. Resolving a complete picture of platinum-resistance has been impeded by lack of rigorous approach in models used, particularly for OVCA. A popular method has been to leverage isogenic platinum-resistant cell lines. However, the two most commonly used OVCA cell lines in the isogenic platinum resistance literature, SKOV3 and A2780, have been determined to be poor models of HGSOC and are likely ovarian clear cell carcinoma (OCCC) and endometrioid ovarian carcinoma (EOC), respectively [11, 12]. The alternative approach, to use clinical specimen data to compare to survival outcomes, is challenged by two problems. First, findings of platinum response in clinical specimens have been predominated by the presence or absence of immune cells in the specimen, rather than more nuanced changes within the tumor. This has been a contributor to subtyping efforts that have revealed that poor clinical responses observed in epithelial-to-mesenchymal transition (EMT)–high subtypes are attributed by an increase in anti-inflammatory M2 macrophages and a lack of anti-tumor T- and NK-cells [13]. This largely masks cell-intrinsic factors that contribute to resistance. Second, most publicly available data available is from patients at initial diagnosis, where most patients are responsive to chemotherapy. Thus, they are not indicative of the changes that occur later in the tumor to make them no longer responsive at a later recurrence.

To more robustly leverage cell lines for the examination of platinum-response predictors, we must first have a more robust understanding of baseline sensitivity of OVCA cell lines to platinum-based chemotherapy. Our study sought to define the platinum sensitivity of 36 OVCA cell lines representing a variety of histotypes, including 11 HGSOC cell lines, and their corresponding genetic and gene expression signatures. Further, we leveraged seven isogenic platinum-resistant cell line pairs derived from four initially platinum sensitive counterparts to more comprehensively determine mechanisms. Our results indicate that common themes of platinum resistance are predominated by innate immunity/STAT activation, epithelial-to-mesenchymal transition (EMT) and stemness, and platinum influx/efflux regulators. This work serves as a resource to the OVCA community for more appropriate and well-defined platinum-responsiveness across multiple OVCA subtypes in the aim that the field of platinum-responsiveness in OVCA can be accelerated by using more appropriate model systems.

## Methods

### Cell culture

OV90 (American Type Culture Collection [ATCC]), TOV112D (ATCC), TOV21G (ATCC) were cultured in a 1:1 mix of MCDB 105 (117-500 Sigma) supplemented with 1.5 g/L NaHCO₃ (ThermoFisher Scientific) and Medium 199 (BW12-119 Fisher Scientific) with 15% fetal bovine serum (FBS; 89510-186 VWR). COV362 (European Collection of Authenticated Cell Cultures [ECACC]) and COV434 (ECACC) were cultured in Dulbecco Modified Eagle’s Medium (DMEM; 10566024 ThermoFisher Scientific) with 10% FBS. CAOV3 (ATCC) were cultured in DMEM + 10% FBS with 1 mM Sodium Pyruvate (NaP; ThermoFisher Scientific). 59 M (ECACC) and OAW42 (ECACC) were cultured in DMEM with 10% FBS, 1 mM NaP, and 2ug/mL human insulin (ThermoFisher Scientific). JHOC5 (RIKEN BioResource Research Center), JHOS2 (RIKEN), and JHOS4 (RIKEN) were cultured in DMEM/Ham’s F-12 with 10% FBS and 0.1 mM non-essential amino acids (NEAA; ThermoFisher Scientific). JHOC9 (RIKEN) were cultured in DMEM/Ham’s F-12 with 20% FBS and 0.1 mM non-essential amino acids (NEAA; ThermoFisher Scientific). RMGI (Japanese Collection of Research Bioresources Cell Bank [JCRB]) were cultured in Ham’s F-12 with 10% FBS. ES2 (ATCC) were cultured in McCoy’s 5 A (12330031 ThermoFisher Scientific) with 10% FBS and 25 mM 4-(2-hydroxyethyl)-1-piperazineethanesulfonic acid (HEPES; Sigma). OVK18 (RIKEN), TYKnu (JCRB), and TYKnu.CPr (JCRB) were cultured in Minimum Essential Medium (MEM; 11095080 ThermoFisher Scientific) with 10% FBS. BIN67 (a generous gift from Barbara VanderHyden, Ottawa Hospital Research Institute), CAOV4 (ATCC), IGROV1 (National Cancer Institute Division of Cancer Treatment and Diagnosis Tumor Repository [NCI DCTD]), KURAMOCHI (JCRB), OVCAR8 (NCI DCTD), OVISE (JCRB), OVMANA (JCRB), OVSAHO (JCRB), OVTOKO (JCRB), and SKOV3 (NCI DCTD) were cultured in RPMI (11875119 ThermoFisher Scientific) with 10% FBS. PEA1 (ECACC), PEA2 (ECACC), PEO1 (CancerTools.org), PEO4 (ECACC), PEO6 (ECACC) were cultured in RPMI 1640 with 10% FBS and 1 mM NaP. OVCAR4 (Millipore-Sigma) was cultured in RPMI 1640 with 10% FBS and 2ug/mL human insulin. EFO21 (DSMZ-German Collection of Microorganisms and Cell Cultures [DSMZ]) and EFO27 were cultured in RPMI 1640 with 15% FBS, 0.1 mM NEAA, and 1 mM NaP. OVCAR3 (ATCC) were cultured in RPMI 1640 with 15% FBS, 10 mM HEPES, 1 mM NaP, and 2ug/mL human insulin. Cell lines used in this study and the media preparations used for culture are also described in Supplemental Table 1. Regarding sample size, we maximized the number of cell lines we could receive from commercial sources for which there was substantial literature supporting ovarian origin and to represent ovarian cancer subtypes.

All cell lines were cultured in a humidified 37°C incubator with 5% CO_2_. Culture medium was supplemented with 1% penicillin/streptomycin (Gibco; Waltham, MA, USA). STR profiling for authentication was performed through the University of Wisconsin-Madison’s Translational Research Initiatives in Pathology (TRIP) Lab. Mycoplasma testing was performed using the MycoAlert^TM^ Mycoplasma Detection Kit (Lonza; Basel, CHE).

### Drug dose response assay

Cells were plated in a white opaque-walled 96-well tissue culture plate (Corning; Corning, NY, USA) at densities indicated in Supplemental Table 1, which ranged from 1000 to 10,000 cells, with a median of 2500 used. Cells were treated with cisplatin (Cayman Chemical, cat. 13119; Ann Arbor, MI, USA) and carboplatin (Cayman Chemical, cat. 13112) 24-hours after plating. A 10-point dose range between 400uM to 60 nM was used for cisplatin and 500uM to 980 nM was used for carboplatin to best capture the point of inflection in survival. 72 hours after adding drugs, CellTiter-Glo 2.0 (Promega, Madison, WI, USA) assay was performed according to the manufacturer’s instructions. Luminescence was read using a BioTek Cytation 5 Cell Imaging Multi-mode Reader (Agilent; Santa Clara, CA, USA). The cisplatin and carboplatin IC_50_ were determined as previously described [14]. Each experiment was performed in technical triplicate (3 wells per dose per cell line) and independent replicates. Additional independent replicates were performed if IC_50_ values between replicates differed by more than 2-fold.

### Copy number signature analysis

Copy number signatures based on signatures in Catalogue Of Somatic Mutations in Cancer (COSMIC) copy number variation signatures version 3.4 were calculated as previously described [15]. We applied this to publicly available copy number data for all OVCA cell lines from the Cancer Cell Line Encyclopedia (CCLE) project, as obtained from DepMap from the ABSOLUTE copy number data (CCLE_ABSOLUTE_combined_20181227). HS178T and OC316 were removed due to known contamination of these samples with other cell lines. The CCLE data was input into SigProfilerMatrixGeneratorR (v1.2) [16], which produces a mutational matrix for the set of samples. This matrix was then the input for SigProfilerAssignmentR (v0.0.23) [17] using the cosmic_fit() function. After this, copy number signatures 1 through 24 from the COSMIC were grouped based on similar etiology as follows: changes in ploidy, chromothripsis associated amplification, focal loss of heterozygosity, chromosomal loss of heterozygosity, tandem duplication and homologous recombination deficiency, unknown, and profile oversegmentation. Weighted and unweighted copy number signatures were plotted; weighted signatures were generated by summing the unweighted scores and adjusting the fraction of contributions of each score out of 100% of the sum.

### Oncoprints

Mutation and gene copy number data was obtained from cBioPortal using the “CCLE Broad, 2019” dataset. Ovarian cell lines were selected for plotting using cBioPortal’s OncoPrinter tool. Genes selected were known homologous recombination deficiency genes (from review [18]). For the associated supplemental table, known allele fractions were obtained from DepMap for single nucleotide polymorphisms, as well as amplifications and deletions. Custom tracks were imported into the OncoPrinter tool, including our cisplatin and carboplatin IC_50_ values, fraction copy number altered and scarHRD scores (as described below).

### scarHRD

Homologous recombination deficiency (HRD) scores were calculated using the scarHRD R package (v0.1.1) [19]. ABSOLUTE copy number data (CCLE_ABSOLUTE_combined_20181227) was obtained from DepMap. scar_score() was run with the following parameters: reference = “grch37”, seqz=FALSE, ploidy = TRUE, chr.in.names = FALSE. The sum of the 3 HRD scores was used as the “scarHRD” score.

### Ploidy and Fraction Copy Number Altered

Ploidy values for cell lines were obtained from DepMap ABSOLUTE analysis from the 20181227 dataset of the CCLE cell lines. Segtab data was used to calculate fraction copy number altered, as follows: sum of the length of regions with copy number alterations that Modal_Total_CN deviated from a value of 2 over the length of all regions measured.

### Generation Of Cisplatin Resistant OVCAR3 and OVCAR4 Cell Lines

OVCAR3 and OVCAR4 isogenic platinum-resistant cell lines were generated by treating each cell line with 1uM cisplatin for 4 hours. After treatment, cells were provided with fresh medium and allowed to recover until the cells resumed their typical growth rate for at least two passages (typically about 2 weeks). The same treatment process was then repeated at the same concentration, and after recovery of the second treatment, the cisplatin dose was increased by 1uM and the process repeated. This process was continued over a period of 9 months. Two independent platinum resistant cell lines were generated for both the OVCAR3 and OVCAR4 lines designated ResA or ResB. The cells are not maintained in cisplatin containing medium. All experiments on OVCAR3 and OVCAR4 resistant lines were performed within 42 days of last measurement of platinum resistance by drug dose response, overall at a median of 10 days following last measurement.

### Flow Cytometry

Cells were harvested using PBS, pH 7.4 + 5 mM EDTA to preserve cell surface proteins. Cells were stained (1:50 dilution) with a CD133-APC conjugated antibody (Miltenyi Biotec, cat. 130-113-184) and ALDH activity assessed using an ALDEFLUOR kit (STEMCELL Technologies; Vancouver, BC, CAN) according to the manufacturer’s protocol. Mouse IgG2b-APC (Miltenyi Biotec, cat. 130-122-932; Bergisch Gladbach, DEU) and single stain controls were performed. Cells were imaged on a CytoFLEX benchtop cytometer (Beckman Coulter; Indianapolis, IN, USA), and data was analyzed using FlowJo. A minimum of two independent replicates were performed for all flow cytometry, and the reported ranges reflect the consensus of the experimental values.

### Tumorsphere Culture

Cells were incubated with tumorsphere medium consisting of Dulbecco’s Modified Eagle Medium/F12 (Gibco), B27 supplement (Gibco), Human Recombinant Basic Fibroblast Growth Factor (STEMCELL technologies), Human Epidermal Growth Factor (PEPROTECH; Waltham, MA, USA), Human Recombinant Insulin (Gibco) and Bovine Serum Albumin (Sigma-Aldrich; St. Louis, MO, USA) in an ultra-low attachment 6-well plate for 1-2 weeks. When tumorspheres were ready to be passaged, the contents of the wells were transferred to a 15 mL conical tube. Cells were incubated for 10–15 min to allow them to settle to the bottom of the tube. The supernatant was removed without disturbing the pellets before 0.05% Trypsin-EDTA was added to break the tumorspheres apart. Media containing serum was utilized to inhibit trypsin activity and the cells were centrifuged. Supernatant was removed, fresh tumorsphere media was added, and the cells were plated in an ultra-low attachment 6-well plate.

### Western Blotting

Cell lines were treated with their respective cisplatin IC_50_s for 72 h before being harvested into pellets. Proteins were extracted using a 9 M urea extraction buffer (9 M urea, 4% CHAPS, 0.5% IPG buffer, 50 mM DTT). Fluorescent detection blots were blocked in 5% skim milk in TBST for 1 hour at room temperature and subsequently probed for a housekeeping gene for 1 hour at room temperature. Following incubation, the blots were probed with a primary antibody for the protein of interest overnight at 4 °C. The blots were incubated with secondary antibodies (LiCor #926-32211 and #926-68070; Lincoln, NE, USA) at 1:20 000 for 1 h at room temperature and scanned on Li-Cor CLx. Primary antibodies: CD133 (Abcam ab19898; 1:1000; Cambridge, MA USA), ALDH1 (BD Biosciences #611195; 1:1000; Franklin Lakes, NJ, USA), Actin (Thermo Fisher Scientific #MA5-15739; 1:5000; Waltham, MA, USA), GAPDH (Cell Signaling Technology #5174S; 1:1000; Danvers, MA, USA).

### Wound Healing Assays

Cell lines were plated in their respective culture media in a 6-well tissue culture plate and were seeded at a density that would reach 70-80% confluence after 24 hours of incubation. The densities used range from 3×10^5^ to 1×10^6^ cells per well depending on the cell line and the rate of growth. Cells attached to the bottom of the plate for 24 hours before staining with 10uM final working concentration of CellTracker Green CMFDA dye (Invitrogen; Waltham, MA, USA). After 30 minutes of incubation, a vertical line was manually scratched into the cell layer, using a 1000uL pipette tip. The scratch was imaged by time lapse microscopy at the same X/Y coordinates every 24 hours using a Cytation 5 using the Biospa robotic attachment at 4x magnification. The width of the scratch was measured in ImageJ for each timepoint using images obtained on the Cytation 5. The person measuring distance was blinded to the timepoint and sample. Three X/Y coordinates per well were used as technical replicates, and experiments were run in 3 or more independent replicates to derive mean and standard deviation. Difference in wound width from the 0 h timepoint for matched image was calculated and expressed as percent wound closure. Unpaired t-test assuming unequal variance was run in Excel, and *p*-values were adjusted by number of comparisons for each cell line.

RNA sequencing preparation and analysis methods are described in the **Supplemental Methods**.

## Results

### Selection of OVCA cell line panel for analysis

We selected a panel of 36 OVCA cell lines for this study, based on representativeness across a wide variety of OVCA histotypes (Supplemental Table 2). We extensively examined the literature on these cell lines, including the original literature on the tumors the cell lines were generated from, where available, and recent genomic, transcriptomic, and proteomic analyses comparing cell lines and tumors of different histotypes to determine the current consensus (Supplemental Table 2). Where literature was uncertain or disagreed, we also consulted DepMap’s Celligner tool to compare cell lines to TCGA tumors, which are predominantly HGSOC [20] (Supplemental Fig. 1A). We made the decision not to include some extensively used cell lines, including A2780 and OVCAR5, as these cell lines have been characterized as potentially problematic [11, 12, 21, 22].

**Fig. 1:**
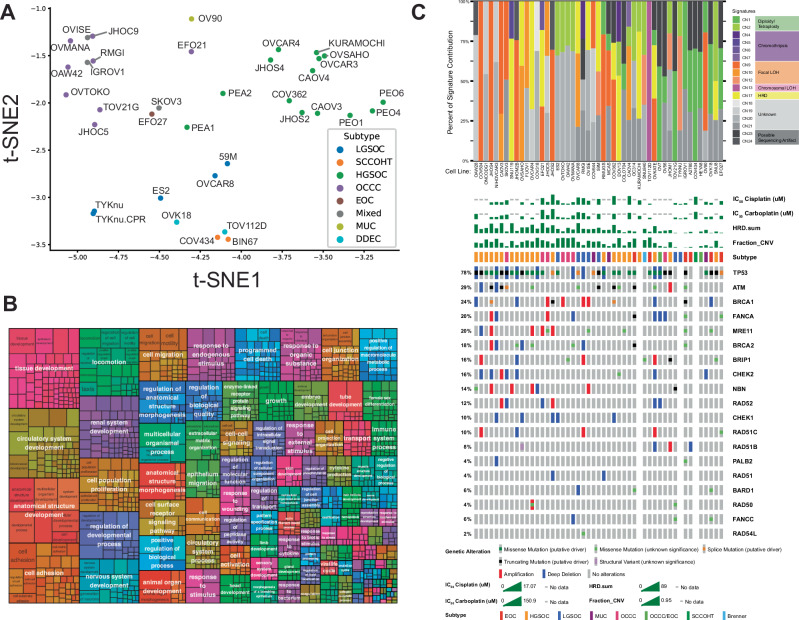
Ovarian cancer cell line gene expression and subtyping and copy number signature analysis. **A** T-distributed stochastic neighbor embedding (t-SNE) plot of cell lines included in the study. Subtype abbreviations are: DDEC = Dedifferentiated Endometrial Carcinoma, EOC = Endometrioid Carcinoma, HGSOC = High-Grade Serous Ovarian Carcinoma, LGSOC = Low-Grade Serous Ovarian Carcinoma, MUC = Mucinous, OCCC = Ovarian Clear Cell Carcinoma, SCCOHT = Small Cell Carcinoma of the Ovary, Hypercalcemic Type. **B** Gene ontology terms for top 1000 most variable genes across the 36 cell lines shown as a tree map. Gene ontology terms were reduced by clustering terms with similarity into parent terms. Size of individual gene ontology terms is based on relative proportion of -log10(*p*_values) weighted across all significant ontology terms plotted. Colors are arbitrary and randomized. **C** Copy number signature analysis and homologous recombination deficiency (HRD) gene oncoprint for OVCA cell lines in CCLE dataset. The stacked bar plot (top panel) summarizes the weighted contributions of each copy number signature to the overall genomic profile. Copy number signatures are colored by shared etiologies, as described in figure legend to the right. The oncoprint (bottom panel) displays known mutations to HRD genes corresponding to the cell lines in the top panel. IC_50_ values for cisplatin and carboplatin in our own assays are also shown as a bar graph, as well as calculated HRD score (HRD.sum) and fraction genome altered (fraction_CNV). Percentages on the left summarize the fraction of cell lines carrying an alteration to each gene. Subtype is derived from our literature review.

We generated RNA-seq data on all cell lines in a single batch of preparation and sequencing to allow direct comparison of gene expression with no batch effects. Subtypes defined based on most current literature estimates are supported by clustering based on similarity of gene expression between the cell lines using t-distributed stochastic neighbor embedding (t-SNE) and principal component analysis (PCA) methods (Fig. 1A, Supplemental Fig. 1B). Top ranked genes that contributed to principal component 1 (PC1), which discriminated HGSOC and OCCC from other subtypes, included genes biologically relevant to epithelial OVCAs: MUC16, PAX8, and many epithelial cell markers (EPCAM, KRT19, KRT7, KRT8, CDH6, CDH1; Supplemental Fig. 1C). Gene ontology terms enriched in the top 1000 most variable genes were condensed into related terms and revealed a predominance of ontologies related to development, cell proliferation/migration/adhesion (Fig. 1B). While OVCAR8, 59 M, and TYKnu have been previously characterized generally as serous/HGSOC cell lines, some literature reports suggest otherwise [12, 23, 24]. Based on distant clustering from TCGA tumors and other HGSOC lines (Supplemental Fig. 1A), the presence of KRAS and ERBB2 mutations in OVCAR8, NRAS mutations in TYKnu, and close clustering with BRAF1 mutant ES2 and HEYA8 cell lines, our data agrees with literature suggesting that they are LGSOC-like.

### Copy number signature analysis

We performed copy number (CN) signature analysis on publicly available genomic copy number data from the Cancer Cell Line Encyclopedia [25] (Fig. 1C). These copy number signatures quantify contributions of copy number by various patterns and pair them to a predicted etiology. Of note, since we leveraged publicly available data, the dataset available for copy number signature analysis does not completely overlap with the cell lines we used in the rest of the study. The most frequent assignable etiologies explaining the observed signatures are related to focal loss of heterozygosity (LOH; CN9, CN10, and CN12), which occurred most frequently in HGSOC cell lines, but also in OCCC cell lines (SKOV3, EFO21, and JHOC5), LGSOC cell lines (OVCAR8, 59 M, and TYKNU), and Mucinous cell lines (MCAS and OV90).

Homologous recombination deficiency (HRD) signatures occurred in twelve cell lines. Seven of these cell lines (58%; SNU119, OVCAR4, 59 M, KURAMOCHI, OVISE, DOV13, and SNU8) lacked biallelic loss in genes attributed to HRD, including BRCA1 and BRCA2 (Fig. 1C and Supplemental Table 3A). HRD signatures were also not restricted to a particular subtype. As an independent measure of HRD for comparison, ScarHRD scores were also applied to the copy number data and generally corroborated HRD called by copy number signature analysis (Supplemental Fig. 2A and Supplemental Table 3A). CN1 corresponds to diploidy, and CN2 corresponds to tetraploidy; these signatures very closely aligned to independently reported ploidy of the cell lines (Supplemental Fig. 2B and Supplemental Table 3B). Chromothripsis signatures were infrequently observed and were limited to the HGSOC and OCCC cell lines SNU119, EFO21, RMGI, DOV13, and CAOV4. Chromosomal LOH was only found in TOV112D, where it was the only attributed copy number signature. These data are low confidence, as TOV112D had the lowest total assignments of copy number signatures (Supplemental Fig. 2C) and has been described to be hyperdiploid [26].

### Sensitivity of ovarian cancer cell line panel to platinum-based chemotherapies

**Fig. 2:**
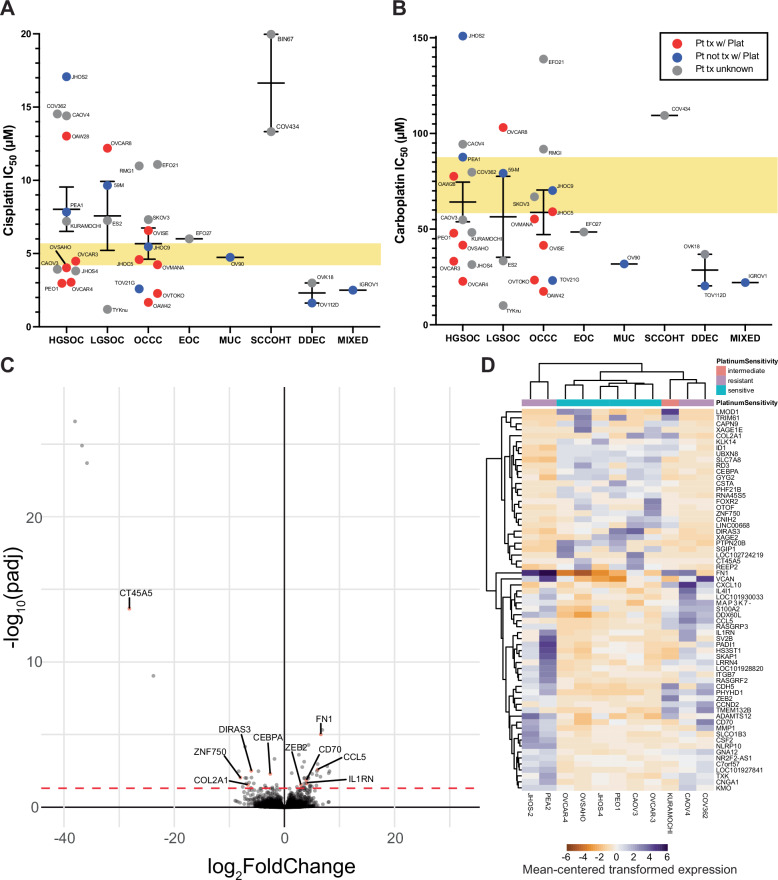
Sensitivity of OVCA cell line panel to platinum-based chemotherapies and resistance-associated gene expression signatures of HGSOC. **A** Cisplatin and (**B**) carboplatin IC_50_ values for 33 OVCA cell lines of various subtypes. Line and error bars for each subtype show the mean +/- standard error of the mean. Colors of points indicate what is known about patient (Pt) pre-treatment with platinum-based chemotherapy prior to derivation of the cell line, where red indicates the Pt received platinum prior to tumor acquisition, blue indicates Pt did not receive platinum, and gray indicates that treatment is unknown. C_max_ range is shown with the yellow rectangle. **C** Volcano plot and (**D**) heatmap showing differentially expressed genes between HGSOC cell lines based on platinum sensitive, intermediate, and resistant classifications from our data in (**A**, **B**). Genes with demonstrated roles in platinum-resistance in the literature are labeled in the volcano plot. Values in the heatmap for each gene are centered on the mean of the transformed expression across the eleven cell lines, and the red dotted line indicates *p* = 0.05.

For our panel of 36 OVCA cell lines, we evaluated the platinum sensitivity of all cell lines by determining the 72-hour IC_50_ values for both cisplatin and carboplatin **(**Fig. 2A, B, Supplemental Table 4A). Since PEO4, PEO6, and PEA2 are cell lines derived from the same patients as PEO1 and PEA1, we did not include them in this summary of platinum-resistance but have included the data in analysis of isogenic cell line pairs below. The highest achievable carboplatin dose did not reduce survival of BIN67 cells enough to calculate an IC_50_. HGSOC, LGSOC, and OCCC subtypes displayed a broad range of sensitivity to platinum reagents, with no statistically significant difference between groups for either cisplatin or carboplatin by t-test. Relative sensitivities to cisplatin and carboplatin were similar. Cell lines were categorized as platinum-sensitive if their 72-hour IC_50_ fell below the clinical C_max_: 58.4-87.4uM for 15–30 minutes of carboplatin infusion at AUC 5–6 [27–29], and 4.1uM and 6.6uM for 1 and 2 hour infusions with cisplatin at 50–80 mg/m^2^ [30–32].

### High-grade serous ovarian carcinoma gene expression associated with resistance

To identify gene expression distinguishing platinum sensitivity from resistance, we specifically examined HGSOC cell lines, as cell line sensitivity varied by subtype, leading to histotype-specific gene expression contributing to resistance signatures when all cell lines considered. Based on the established in vitro sensitivity to cisplatin and carboplatin, we used the HGSOC cell lines subsetted into platinum resistant (JHOS-2, PEA2, CAOV4, COV362), intermediate (KURAMOCHI), and sensitive (OVCAR-3, OVCAR-4, OVSAHO, JHOS-4, PEO1, CAOV3) groups to perform differential expression analysis. PEA1, PEO4, and PEO6 were removed from analysis to avoid over-representation of a single patient that may introduce bias. This resulted in 64 differentially expressed genes, with 37 up-regulated and 27 down-regulated (Fig. 2C, Supplemental Table 4B). A subset of genes were highly expressed in only a subset of resistant or sensitive cell lines, which resulted in clustering of resistant cell lines CAOV4 and COV362 closer to sensitive cell lines (Fig. 2D).

Of the 56 differentially expressed genes, ten of these had some prior evidence of connection to platinum-resistance. We excluded genes associated with adaptive immune responses, as the conditions under which platinum-resistance was examined in our experiments lacked immune components. The five upregulated genes were CCL5, FN1, CD70, IL1RN, and ZEB2, and the five downregulated genes were CEBPA, DIRAS3, COL2A1, ZNF750, and CT45A5.

### Differential expression of HGSOC isogenic platinum-resistant pairs

**Fig. 3:**
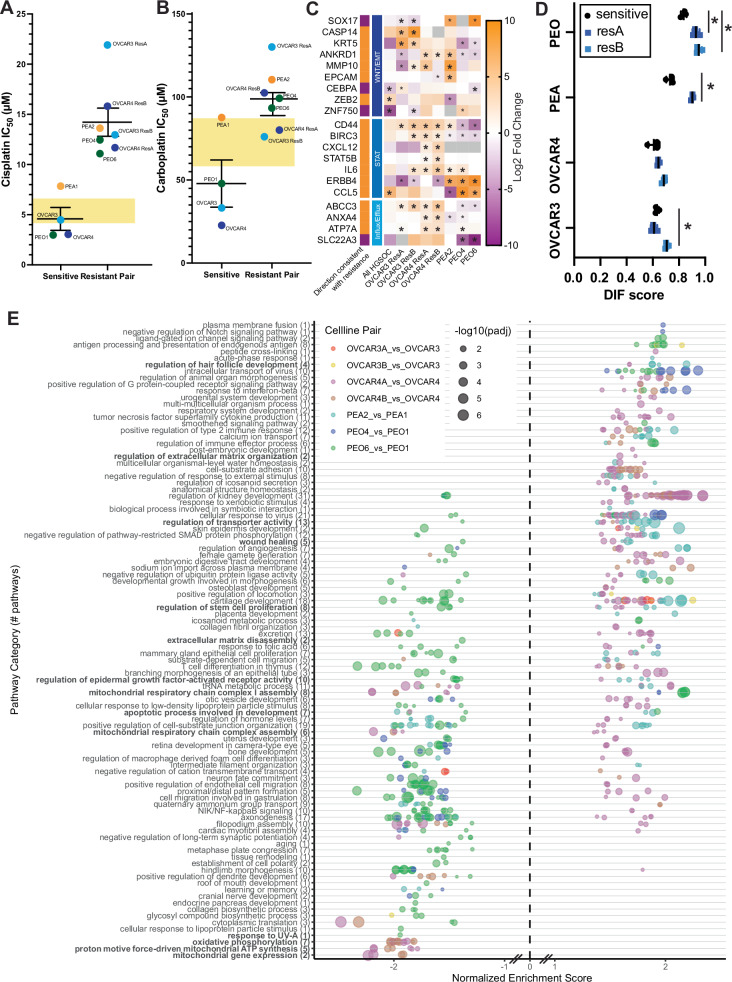
Characterization of isogenic platinum resistant HGSOC cell lines. **A** IC_50_ values for cisplatin and (**B**) carboplatin comparing the parental platinum sensitive cell line (left) to its isogenic resistant pair(s) (right). Line and error bars for the sensitive cell lines and resistant pairs show mean +/- standard error of the mean. C_max_ range is shown with the yellow rectangle. **C** Heatmap of WNT/EMT, STAT, and Platinum Influx/Efflux gene expression from RNA-seq. Genes were selected based on combination of magnitude of change across all differential gene expression datasets and literature relevance to platinum. Expected directionality of change associated with resistance is shown on far left. Asterisks indicate adjusted *p*-values < 0.05, with larger asterisks indicating directionality consistent with resistance. **D** CLOVAR differentiated gene signature score for individual RNA-seq replicates. Asterisks indicate adjusted *p*-value < 0.05 with unpaired t-test. **E** Gene ontology analysis showing overrepresented pathways in the differentially expressed genes found when comparing the sensitive and resistant isogenic pairs individually. Gene ontology results are aggregated by semantic space to shared pathways, where the number of individual gene ontology terms is indicated in the label in parentheses. Each isogenic pair is color-coded independently, as indicated in the legend.

To further explore genes related to platinum resistance, we generated isogenic platinum-resistant pairs of the HGSOC cell lines OVCAR3 and OVCAR4 through pulse-treatment of the cells with cisplatin until the IC_50_ value for the cell line increased at least 3-fold. Two independent isogenic pairs were generated for both cell lines (OVCAR3/4 ResA and ResB). In addition, commercially available isogenic platinum sensitive and resistant HGSOC cell lines derived from the same patient were utilized (PEO1/4/6 and PEA1/2) [33]. Collectively, the resistant pairs showed a 1.7-4.8-fold increase in cisplatin resistance (Fig. 3A, Supplemental Fig. 3, and Supplemental Table 4A) and a 1.3-4.5-fold increase in carboplatin resistance (Fig. 3B and Supplemental Table 4A). We performed RNA-seq on the parental sensitive and isogenic resistant pairs to determine gene expression changes associated with resistance (Supplemental Table 4C).

We layered manually annotated scores from a publicly available database of genes associated with platinum resistance based on the prior literature [10] on volcano plots of gene expression fold change against *p*-value in our isogenic pairs to determine whether there was a predominance of seven key resistance themes (Supplemental Fig. 4): oncogenic signaling, DDR, apoptosis inhibition, extracellular matrix modifications, platinum influx/efflux, metabolic changes, and hypoxia responses. At the gene expression level, oncogenic signaling and extracellular matrix modification contributors to resistance were most strongly modified categories. Generally, individual isogenic resistant cell lines from the same parental cell line had more diversity in gene expression contributions to resistance between individual resistant lines than the clinical pair (PEO4/6), which are thought to originate from a shared clone [34]. Wnt signaling, EMT, intrinsic inflammatory responses, and platinum influx/efflux were predominant across models, which are summarized in Fig. 3C.

We examined whether clinical signatures of response that have been described are also changed in our isogenic models. We ran previously validated prognostic gene signatures, CLOVAR and Oxford Classic, and found that classifications were largely unchanged between lines, and generally lacked classifications related to immune infiltration, as expected. The only notable change observed was in CLOVAR differentiated clusters, where resistant lines tended to have higher differentiated signature scores (Fig. 3D). This was statistically significant for OVCAR3 ResB, PEA2, PEO4, and PEO6. Specific genes in these signatures that were frequently upregulated between sensitive and resistant lines were C1ORF116, MYO5C, PTGER2, DTX4, SLC37A1, and ALS2CL.

**Fig. 4:**
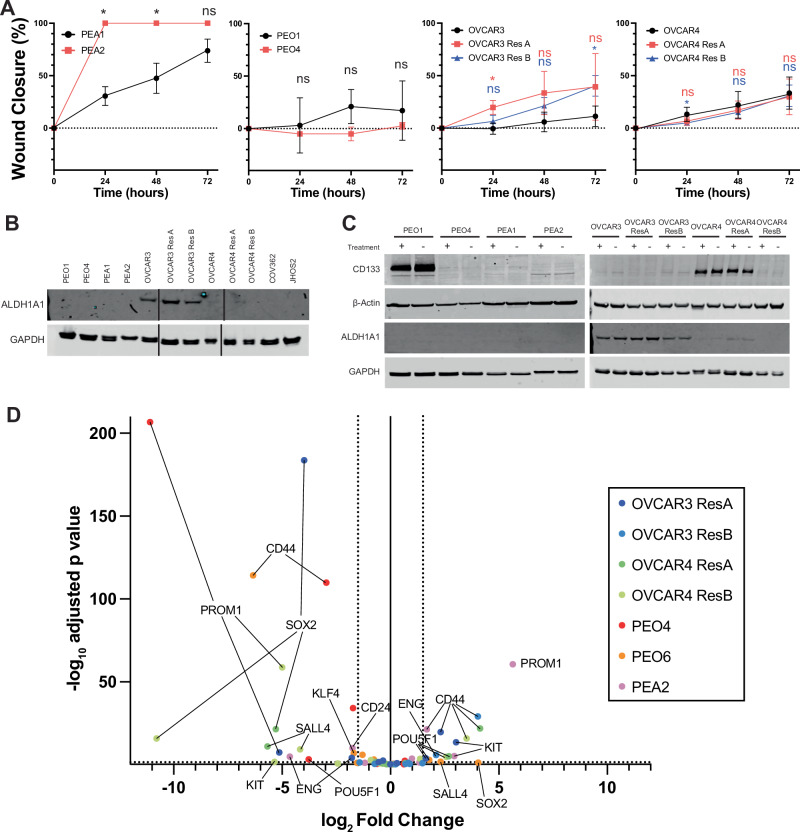
Markers of stemness and wound healing in isogenic platinum resistant HGSOC cell lines. **A** Wound healing assay for the PEA1/PEA2 (*N* = 3 independent replicates), OVCAR3 (*N* = 4), OVCAR4 (*N* = 4), and PEO1/4 (*N* = 3) isogenic cell line pairs. Error bars represent standard deviation. * indicates *p*-value < 0.05 in paired t-tests and ns indicates non-significant *p*-value > 0.05. **B** Representative ALDH1A1 western blot images of all isogenic cell line pairs along with platinum resistant cell lines JHOS2 and COV362. GAPDH serves as a loading control. **C** CD133 and ALDH1A1 western blot images of all isogenic cell line pairs with or without cisplatin treatment at each respective cell line’s IC_50_ for 72 hours. GAPDH serves as a loading control. **D** Volcano plot showing differential expression between isogenic platinum-resistant cell lines, comparing resistant to their sensitive counterpart. Points are color-coded based on the specific pair, and statistically significant ( | log2 fold change |>1.5, adjusted *p*-value < 0.05) genes are labeled by gene name.

Gene ontology analysis revealed several resistance-related mechanisms (Fig. 3E**)**. Many of the identified pathways were not shared across isogenic pairs, and instead were either only found in a few pairs, or were differentially enriched in different pairs. Since wound healing was identified in a few pairs, we performed scratch assays on isogenic platinum-resistant cell lines. Scratch closure was significantly increased for PEA2, OVCAR3 ResA (24 h), OVCAR3 ResB (72 h) (Fig. 4A and Supplemental Fig. 5). The most prominent change was in PEA2, which was consistent with the increase in gene ontology pathway enrichment scores for this cell line (Fig. 3E). OVCAR4 ResB had significant early delay in scratch closure.

### Stemness markers in isogenic resistant pairs

As cancer stem cell or quiescent states have been implicated in the development of resistance to platinum-based chemotherapy, we examined whether the isogenic platinum-resistant cell lines exhibited more stemness markers compared to their sensitive counterpart. Differential expression analysis of isogenic pairs revealed that PEO4 and PEO6 had increased “regulation of hair follicle development”, PEO6 had decreased “regulation of stem cell proliferation”, and PEA2 and OVCAR4 ResA had increased “regulation of stem cell proliferation” (Fig. 3C), suggesting involvement of these pathways in cisplatin resistance in these pairs. We examined expression of the two most reliable markers of OVCA stemness phenotype, CD133 and Aldehyde Dehydrogenase (ALDH) by both western blot and flow cytometry in all our isogenic pairs (Table 1 Fig. 4B, C, and Supplemental Fig. 6). While we anticipated the resistant cell lines having higher expression of these marks, notably, they were nearly always lower in the resistant cell lines compared to their sensitive counterparts. This was also consistent with the overall picture of gene expression associated with stemness in the isogenic lines: overall, while there are statistically significant changes to genes most important for stemness, there were more decreases in resistant derivatives than there were increases (Fig. 4D). CD44 and OCT4 (POU5F1) were the only stem-related genes that were increased in more than one resistant line, but they are also decreased in others. Similarly, resistant lines were generally no better at forming tumorspheres (Table 1). PEO1 had high CD133 expression and ALDH activity, but PEO4 had markedly decreased expression and activity, and could not form tumorspheres. OVCAR3 had higher tumorsphere formation ability than resistant cell line counterparts, which seems to be most correlated with a decrease in CD133 expression for OVCAR3 ResA and a decrease in ALDH activity for OVCAR4 ResB. OVCAR4 resistant clones had partial to complete loss of CD133, but minimal change to ALDH activity, but tumorsphere formation was largely unaffected. PEA2 resistant cells were the only isogenic resistant line to demonstrate increased tumorsphere formation, which correlates with increased ALDH activity in this cell line. Overall, isogenic platinum-resistant cell lines appear to be poor models of stemness as a feature promoting resistance.

**Table 1:** Summary of stemness markers and phenotypes for platinum-resistant isogenic pair cell lines.

Cell Line	Western Blot		Flow Cytometry		Tumorspheres
	CD133	ALDH1	CD133	ALDEFLUOR	
PEO1	+++	−	+++	+++	+++
PEO4	−	−	+	+	−
PEA1	−	−	+	+	+
PEA2	−	−	+	++	+++
OVCAR3	+	+++	+	+	+++
OVCAR3ResA	−	+++	−	+	+
OVCAR3ResB	+	++	+	−	+
OVCAR4	+++	+	+++	+	+++
OVCAR4ResA	++	+	++	+	+++
OVCAR4ResB	+	−	+	+	+++

For western blot, relative expression of CD133 and ALDH1 are shown. For flow cytometry: − less than 2x negative control, + 2x negative control-30% positive cells, ++ 30%-70%, +++ >70%. For tumorspheres: − for no tumorsphere formation, + for no tumorspheres surviving passaging, +++ for tumorspheres forming for at least 2 passages.

For Flow Cytometry:

− : lower than 2x negative control.

+ : 2x negative control - 30%.

+ + : 30-70%.

+ ++ : higher than 70%.

## Discussion

OVCA cell lines have long been used to study therapeutic response, yet a comprehensive examination of platinum-response in equivalent experimental setup has not been robustly reported in this many cell line models. To our knowledge, only one other study has examined platinum sensitivity at this scale, but only reported visualization of relative sensitivity in a heatmap format and did not contextualize sensitivity to clinically achievable doses [35]. In our study, IC_50_s generally diverged into values that were either above or below the C_max_ of cisplatin and carboplatin. Thus, we are confident that there is a clinically translatable resistance level in the models we have examined. Interestingly, HGSOC and LGSOC displayed a wide range of sensitivity, with cell lines spanning platinum doses above and below the C_max_. EOC, OCCC, Mixed, and DDEC cell lines were largely sensitive to platinum. SCCOHT cell lines were resistant to platinum, consistent with the clinical observations that high-dose mulitidrug chemotherapy regimens are most effective for these patients [36].

**Fig. 5:**
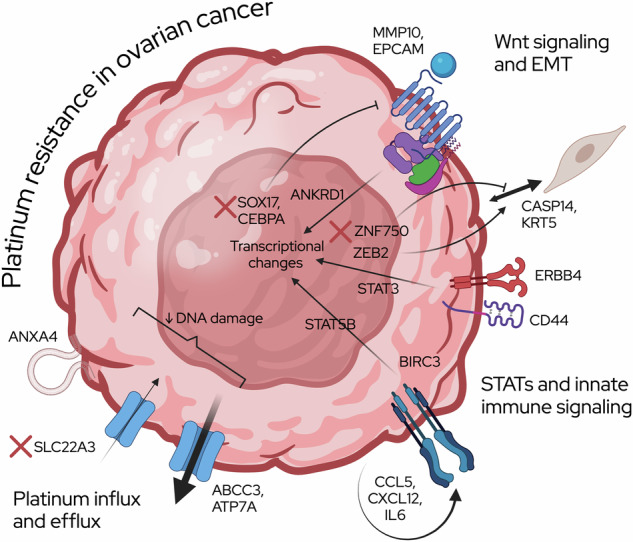
Summary Figure. Summary of findings on platinum resistant HGSOC.

We also used an expanded isogenic HGSOC platinum-resistant pair panel, including three commercially available clinically platinum resistant models [33], and four novel cisplatin-resistant derivatives of OVCAR3 and OVCAR4. Pulse-treatment with cisplatin was used to most closely emulate the selection pressures observed with treatment in patients [37]. We found a predominance of innate immunity/STAT activation, EMT, and platinum influx/efflux regulators in the differentially expressed genes between resistant and sensitive cell lines (Fig. 5). Wnt signaling and EMT related gene expression changes were predominantly found in OVCAR3 ResB, OVCAR4 ResA and PEA2. SOX17 and CEBPA, which are decreased in our models, inhibit WNT signaling [38, 39], and SOX17 negatively regulates the expression of DDR genes [40]. ANKRD1 is a target of TGF-beta and WNT signaling [41]. Matrix metalloproteinase MMP10 is over-expressed in platinum-resistant A2780 cells [42] and can activate canonical Wnt signaling [43]. EPCAM, an epithelial adhesion protein associated with EMT, has an intracellular domain shown to induce Wnt signaling. Upregulation of CASP14 and KRT5 is associated with keratinization. Transcriptional repressor ZEB2 has been associated with EMT in OVCA [44] and is re-expressed in models of chemoresistant OVCA [45]. In contrast, the downregulated ZNF750 transcription factor is a marker of epithelial phenotypes, suggesting a role in EMT.

Changes to platinum influx and efflux were frequent in OVCAR3 ResB, OVCAR4 ResA, OVCAR4 ResB, PEO4, and PEO6. ABCC3 is a well-known multidrug transporter channel involved in the efflux of many drugs out of cells. ATP7A is a copper-transporting ATPase that can mediate resistance to platinum agents [46, 47]. ANXA4, or Annexin A4, promotes membrane fusion and exocytosis and is involved in platinum resistance in OCCC [48, 49]. Cation transporter SLC22A3 is involved in influx of cisplatin into cell, but may not transport carboplatin [50–52].

Increases in STAT signaling and innate inflammation were mostly found in OVCAR3 ResB, OVCAR4 ResA, OVCAR4 ResB, PEO4, and PEO6, and many genes have been implicated in platinum response in OVCA and other cancers [53–63]. CD44, a hyaluronic acid receptor, is a cancer stem cell marker and activates STAT3. Through autocrine signaling, IL6 upregulates STAT3/HIF-1alpha [64], and also induces BIRC3, which regulates NF-kB activation and anti-apoptotic caspase regulation in OVCA cells [65]. BIRC3 levels are high in platinum-resistant A2780 cells [66, 67]. Chemokine CXCL12 treatment of SKOV3 induces cisplatin resistance [68] and induces WNT/beta-catenin and EMT [69] and JAK2/STAT3 [60]. STAT5B is activated downstream of ERBB4 and IL-11 in platinum resistance [61, 62]. ERBB4 can also activate STAT3 [63]. Chemokine CCL5 increases cancer stemness features [70], and STAT3 and PI3K/AKT activation [53].

While we anticipated seeing that resistant cells would have higher positivity for markers of stemness, this was generally not the case, indicating that stemness phenotypes must be a transient state through which resistant cells pass, not a selected trait, consistent with the persister cell hypothesis [71]. Notably, despite the lack of clear canonical markers of stemness phenotype and function, we do see some gene expression changes to EMT and stemness genes that may contribute to resistance. Our overall conclusion from the stemness assays is that isogenic platinum-resistant cell lines are not a good model of the transitory state of platinum resistance development but represent the states that likely exist in a steady-state resistant population.

Our study also builds on work done by others to confirm the histotype of OVCA cell lines [11, 12, 22, 23, 35]. Domcke et al. have presented the largest study of cell lines to date (*N* = 47), where they classified cell lines on likelihood of being HGSOC vs alternatives, largely on genomic features (*e.g*., copy number alterations, tumor mutation burden, and select somatic variants) from publicly available data [11]. Our work builds on this work in a few ways. First, this study focused on histotype-specific gene expression. While Domcke et al. included gene expression data, it was run using simple hierarchical clustering, and was not performed in a single batch as was done in our study. Our use of t-SNE for clustering performed better than hierarchical clustering for distinguishing subtypes (data not shown), likely due to the dimensionality of the data and the number of histotypes represented. Our study also took larger efforts to define non-HGSOC by histotype. In addition, we added demonstrate the first copy number signature analysis of OVCA cell lines, and have added analysis of cell lines JHOC9, PEO1/4/6, PEA1/2, TOV112D, and BIN67.

We note some limitations of the design of our study. While we made attempts to compare the cell lines representing diverse subtypes of OVCA to clinical tumor RNA-seq data, a comprehensive dataset including a representative set of each of these tumors was not publicly available. Inspired by CASCAM [72], we attempted to batch correct several subtype-specific datasets and integrate with our own data using and ComBat, but we found that batch correction largely removed features that defined subtypes. Future studies with RNA-seq data evenly representing different OVCA subtypes would aid in building a foundation upon which we could better assess cell lines against patient tumor subtypes.

Our data suggest multi-mechanism contributions to resistance even within a single resistant cell line that continue to be a challenge to tease apart. Future research must be done to examine which genes contribute most to resistance, or if certain sets of genes work additively or synergistically to modify platinum response. Yet, the robustness of the cell lines chosen and the number of isogenic pairs examined are strengths compared to similar studies on gene expression changes with resistance in OVCA cell models, which have mostly relied on A2780 and SKOV3 derivatives that do not represent HGSOC. Further, applying the differential gene expression changes to a clinical transcriptomic dataset with well-annotated platinum-free intervals (PFI) would greatly improve the biomarker implications of our findings, but even TCGA lacks robust numbers of tumors with both RNA-seq and PFI information available. In the future, we plan to build on our gene expression networks through the integration of shared master regulators and epigenetic organization to develop a more integrated picture of upstream targets with pleiotropic effects that converge to promote resistance.

In summary, we provide a resource of platinum responsiveness data for over thirty ovarian cancer cell lines of different histological subtypes paired with gene expression profiles that support histotype classifications and point to potential mechanisms of resistance. We further examined a set of seven isogenic pairs of platinum-sensitive and -resistant HGSOC pairs to increase our power to detect genes contributing to chemotherapy response in a cell autonomous manner. Overall, we most consistently found themes of innate immunity and STAT activation, EMT and Wnt signaling, and platinum influx and efflux as likely major contributors to HGSOC platinum resistance (Fig. 5). Future work must be done to further test multi-gene contributions to platinum resistance and performance on clinical ovarian cancer samples.

## Supplementary information

Supplemental Materials and Methods

Supplemental Table 1

Supplemental Table 2

Supplemental Table 3

Supplemental Table 4

## Data Availability

Gene expression data is deposited at GEO under accessions GSE273995 and GSE274006.

## Code availability

Code used for our analysis is deposited at our GitHub repository: https://github.com/jessicalanglab/ovarian_cancer_cisplatin_response_manuscript/.
