# Supplementary material for: Cell-intrinsic platinum response and associated genetic and gene expression signatures in ovarian cancer
Source: Cancer Gene Ther. 2025 Jul 19;32(9):985–96. doi: 10.1038/s41417-025-00941-5 (PMC12396967; doi:10.1038/s41417-025-00941-5)

Supplemental materials

Supplemental methods

RNA-sequencing

Total RNA was extracted from cell pellets using a Quick DNA/RNA Miniprep Plus Kit (Zymo Research) per manufacturer's instructions with DNase digestion. RNA quality was assessed using RNA High Sensitivity ScreenTapes and Reagents and analyzed on a TapeStation 4200 instrument (Agilent) and quantified using a Qubit™ RNA Broad Range Kit (Thermo Fisher Scientific). cDNA library prep was performed using the KAPA mRNA HyperPrep Kit (Roche) according to manufacturer's directions with 300ng input and the following modifications: RNA fragmentation at 94°C for 6 minutes, 10 cycles of PCR amplification. Libraries were QC'd using a Qubit™ 1x dsDNA High Sensitivity Kit and High Sensitivity D1000 ScreenTapes. Libraries were equimolarly pooled to 750pM loading concentration. Sequencing was performed on a NextSeq 1000 P2 100 cycle cartridge according to Illumina protocols. For all RNA-seq experiments, the same library pool was sequenced over multiple runs to achieve the necessary level of sequencing depth.

RNA-sequencing analysis

BCL to FASTQ conversion was performed on instrument using Illumina's DRAGEN DRAGEN BCL Convert v3.8.4 for NextSeq 1000/2000. FASTQ files were then used as input into the NextFlow (v22.04.5[1]) pipeline nf-core/rnaseq pipeline (v3.8.1[2]) using genome GRCh38 for alignment and annotation. From pipeline reports of sequencing QC, aligned reads ranged from 25.7 - 40.6 million paired-end reads (mean: 34.4M, standard deviation +/- 3.3M).

Subsequent analysis was performed on the Salmon gene count matrix. T-SNE was used for nonlinear visualization of the gene counts across cell lines. The Salmon gene count matrix was transformed using DESeq2's median of ratios normalization technique[3]. After performing a log

transform and standardization of the normalized counts, the top 1000 variable genes were plotted using sklearn's implementation of t-SNE.

Gene enrichment analysis of the GO pathways for the top 1000 variable genes was performed using gprofiler[4,5]. This was followed by Revigo clustering of the enriched GO pathways[6]. Principal component analysis (PCA) was performed with the DESeq2::plotPCA command on Salmon counts transformed using DESeq2::vst to determine if any likely sample swaps existed, as well as to confirm the consensus subtypes inferred from the literature. Data was plotted with ggplot2.

Differential analysis was performed separately for each sensitive/resistant pair of cell lines, using DESeq2::DESeq with model design ~ Cell line + Replicate. Genes that were differentially expressed with an adjusted p value < 0.05 were designated as differentially up/down regulated genes. Subsequently, gene ontology was performed using clusterProfiler to determine which of the GO biological pathways were overrepresented in the differentially expressed genes (considering up/down regulated genes separately). Finally, Revigo's rrvgo package was used to cluster and summarize the overrepresented pathways.

Gene signatures: After transforming the gene expression values using DESeq2's median of ratios normalization technique, each OVCA cell line was compared to the CLOVAR subtype gene signature, which is a gene signature predictive of HGSC subtype[7]. Comparison with this gene signature results in four scores per OVCA cell line, one for each of the four CLOVAR-annotated subtypes: mesenchymal, proliferative, differentiated, and immunoreactive. The TumorDecon python package was used to perform this analysis, using the parameters specified in the original CLOVAR paper [8]. This consisted of using ssGSEA with an alpha value of 0.75 as the deconvolution method followed by normalization based on random permutations of the gene rankings[7]. A linear regression analysis was conducted to investigate the relationship between IC_{50} values and CLOVAR differentiated scores across the HGSC samples. This analysis showed a positive correlation between IC_{50} and CLOVAR differentiated score, with $p < 0.05$. To further investigate this relationship, the same deconvolution techniques were applied to the dataset of isogenic cell lines. A mixed-effects model

was used to explore the association between resistance and the CLOVAR differentiated score, using a discrete resistant/sensitive annotation and accounting for the grouping of cell lines by isogenic line. This analysis showed that resistant classification was associated with a 12% higher CLOVAR differentiated score, with $p < 0.001$.

Method References

1. Di Tommaso, P. *et al.* Nextflow enables reproducible computational workflows. *Nat Biotechnol* **35**, 316–319 (2017).
2. Ewels, P. A. *et al.* The nf-core framework for community-curated bioinformatics pipelines. *Nat Biotechnol* **38**, 276–278 (2020).
3. Love, M. I., Huber, W. & Anders, S. Moderated estimation of fold change and dispersion for RNA-seq data with DESeq2. *Genome Biol* **15**, 550 (2014).
4. Kolberg, L. *et al.* g:Profiler—interoperable web service for functional enrichment analysis and gene identifier mapping (2023 update). *Nucleic Acids Research* **51**, W207–W212 (2023).
5. Thomas, P. D. *et al.* PANTHER : Making genome-scale phylogenetics accessible to all. *Protein Science* **31**, 8–22 (2022).
6. Supek, F., Bošnjak, M., Škunca, N. & Šmuc, T. REVIGO Summarizes and Visualizes Long Lists of Gene Ontology Terms. *PLoS ONE* **6**, e21800 (2011).
7. Verhaak, R. G. W. *et al.* Prognostically relevant gene signatures of high-grade serous ovarian carcinoma. *J Clin Invest* **123**, 517–525 (2013).
8. Le, T., Aronow, R. A., Kirshtein, A. & Shahriyari, L. A review of digital cytometry methods: estimating the relative abundance of cell types in a bulk of cells. *Brief Bioinform* **22**, bbaa219 (2021).

Supplemental Table and Figure Legends

Supplemental Table 1. Cell line sources, culture conditions, and assay cell densities for each cell line used in our study.

Supplemental Table 2. Summary of cell line subtype classification from literature review. *Detail not found in published data but stated in information provided by the cell line supplier. Literature summaries of performance in spheroid cultures and mouse xenografts is also summarized.

Supplemental Table 3. Homologous recombination deficiency (HRD) scores and ploidy estimates for OVCA cell lines. In the HRD tab, the scarHRD HRD score is reported as HRD.sum. Presence of copy number signatures related to HRD are reported as a value of 1 in the "HRD CN signature". Mutations to common HRD-related genes are also specified by gene, where numeric values indicate the known allelic fraction. In the ploidy tab, ploidy indicates the value from CCLE data. Presence of copy number signatures related to genome doubling and diploidy/tetraploidy are shown as a value of 1. Fraction genome altered is also reported based on CCLE data.

Supplemental Table 4. (A) Cisplatin and Carboplatin IC_{50} values for each cell line used in the study, including isogenic platinum resistant cell lines created in our laboratory. All values are from a 72 hour assay. (B) Differential gene expression analysis of HGSOC cell lines, comparing resistant cell lines to sensitive cell lines, such that a positive log2 fold change indicates higher expression in resistant cell lines. (C) Differential gene expression analysis of isogenic platinum-resistant pairs, such that a positive log2 fold change indicates higher expression in the resistant cell line compared to their sensitive parental counterpart.

Supplemental Figure 1. Principal component analysis (PCA) on gene expression across 36 OVCA cell lines included in the study. (A) DepMap Celligner plot of cell lines (circles), TCGA tumors (+), and other samples such as PDXs (triangles), where distance of points is based on similarity of gene expression of samples. Colors are based on the subtype currently defined in DepMap, not based on our annotations. Inset shows clusters of cell lines in which OVCAR8, 59M, and TYKnu are highly related. (B) PCA plot of the first two principal components (PC1 and PC2). Color coding represents the literature-supported subtype classifications. Subtype abbreviations are: DDEC = Dedifferentiated Endometrial Carcinoma, EOC = Endometrioid Carcinoma, HGSOC = High-Grade Serous Ovarian Carcinoma, LGSOC = Low-Grade Serous Ovarian Carcinoma, MUC = Mucinous, OCCC = Ovarian Clear Cell Carcinoma, SCCOHT = Small Cell Carcinoma of the Ovary, Hypercalcemic Type. (C) Top 20 contributing genes to each of the first two PCs. Loadings were ranked by absolute value for each PC, and the corresponding loading rank for each gene in the reciprocal principal component is shown.

Supplemental Figure 2. Association of between homologous recombination deficiency (HRD) scores and ploidy estimates for OVCA cell lines. (A) ScarHRD scores are plotted for each OVCA cell line in CCLE, then labeled blue if the cell line also had a homologous recombination-related copy number signature etiology associated with it. (B) Ploidy estimates from CCLE for OVCA cell lines adjusted such that 0 indicates diploidy are plotted. Bars are colored light green for presence of tetraploidy copy-number signatures (CN2, CN10, CN12) and dark green for presence of diploidy copy-number signatures (CN1, CN9). Unweighted copy number signature contributions for each cell line. (C) Copy number signature analysis for OVCA cell lines in CCLE dataset. The stacked bar plot is the unweighted equivalent of Figure 2. Copy number signatures are colored by shared etiologies, as described in figure legend to the right.

Supplemental Figure 3. Isogenic platinum-resistant pair drug response and gene expression

relatedness. Cisplatin drug dose response curves. Error bars represent standard error of the mean across 3-4 independent replicate experiments, each with 3 technical replicates.

Supplemental Figure 4. Volcano plots showing differentially expressed genes in the platinum sensitive/resistant isogenic cell line pairs divided into categories related to platinum resistance. Based on previous literature review of genes associated with platinum-response[12], genes were categorized based on seven main, non-mutually exclusive mechanisms of resistance, which are plotted individually. Point size indicates the strength of evidence for contribution to platinum resistance, and points are colored green if directionality of change with resistance is positively correlated with the log2-fold change observed in our data, and red if directionality of change was negatively correlated (opposite direction) as the log2-fold change observed in our data.

Supplemental Figure 5. Representative images of a scratch wound healing assay for the PEA1/2 isogenic cell line pair. Red lines indicate the manually annotated scratch boundary, where it could be found.

Supplemental Figure 6. Representative flow cytometry data for CD133 and ALDEFLUOR (ALDH activity) for each isogenic cell line pair. DEAB is an inhibitor of ALDH enzymes that serves as a baseline negative control in the ALDEFLUOR assay. Negative control for CD133 stain is isotype control. Plot shown were first gated on live cell population.

Supplemental Figure 1

A

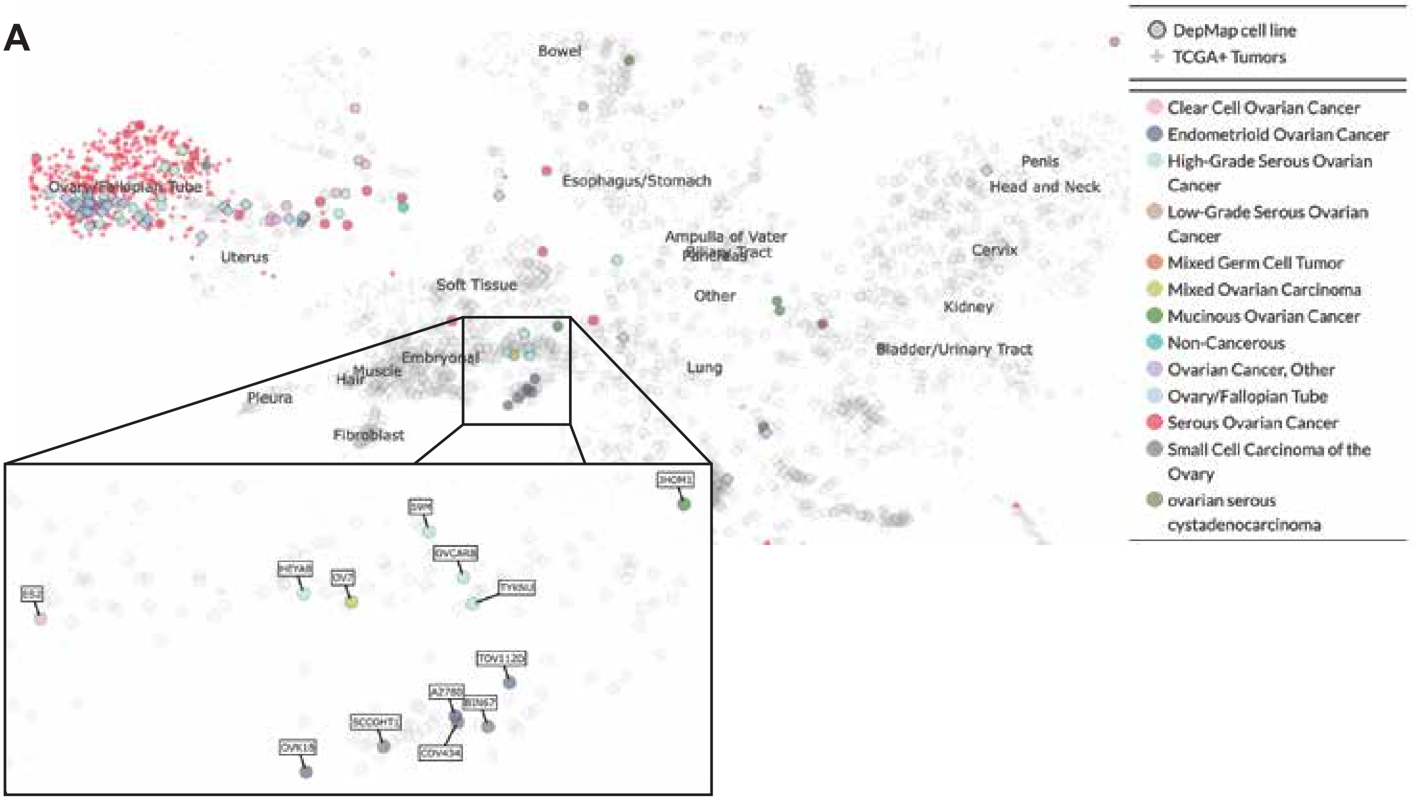

B

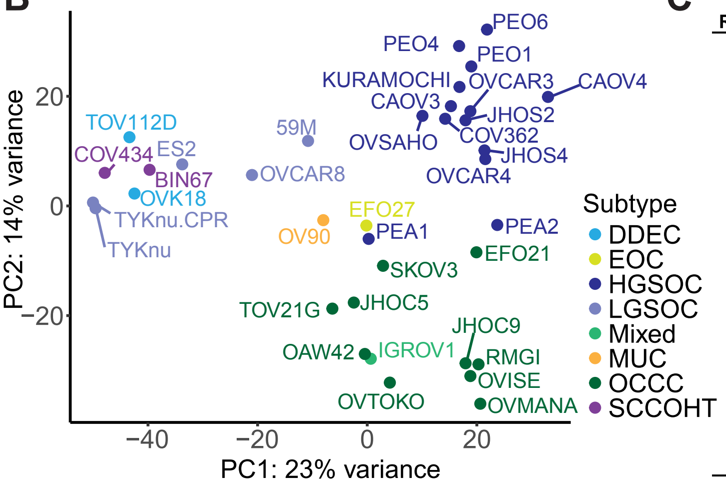

C

Rank	Gene	PC1 Loading	Rank in PC2	Gene	PC2 Loading	Rank in PC1
1	MAL2	0.115	478	SPP1	-0.156	285
2	SPON1	0.104	211	VCAN	-0.144	352
3	LAMA3	0.104	378	HAVCR1	-0.123	341
4	KRT19	0.104	490	HNF1B	-0.121	273
5	MUC16	0.101	26	NID2	-0.119	89
6	EPCAM	0.098	461	RXFP1	-0.107	223
7	DSP	0.097	450	SPOCK2	0.106	28
8	KRT7	0.096	180	PADI1	-0.100	262
9	KRT8	0.095	455	SPARC	0.099	270
10	CD24	0.091	130	FBN2	0.096	458
11	ANXA3	0.090	305	LOC102724788	-0.096	371
12	CDH6	0.090	13	PBX1	0.095	237
13	TACSTD2	0.089	46	CDH6	0.094	12
14	UCA1	0.088	500	KLK5	0.091	158
15	PAX8	0.088	217	UQCRH	0.091	209
16	SLPI	0.087	463	KCNJ16	-0.090	311
17	ESRP1	0.086	289	SCIN	-0.087	475
18	CDH1	0.086	446	ITGB3	-0.086	219
19	EHF	0.086	494	COL4A1	-0.086	55
20	FAM84B	0.085	335	NOS1	-0.084	178

Supplemental Figure 2

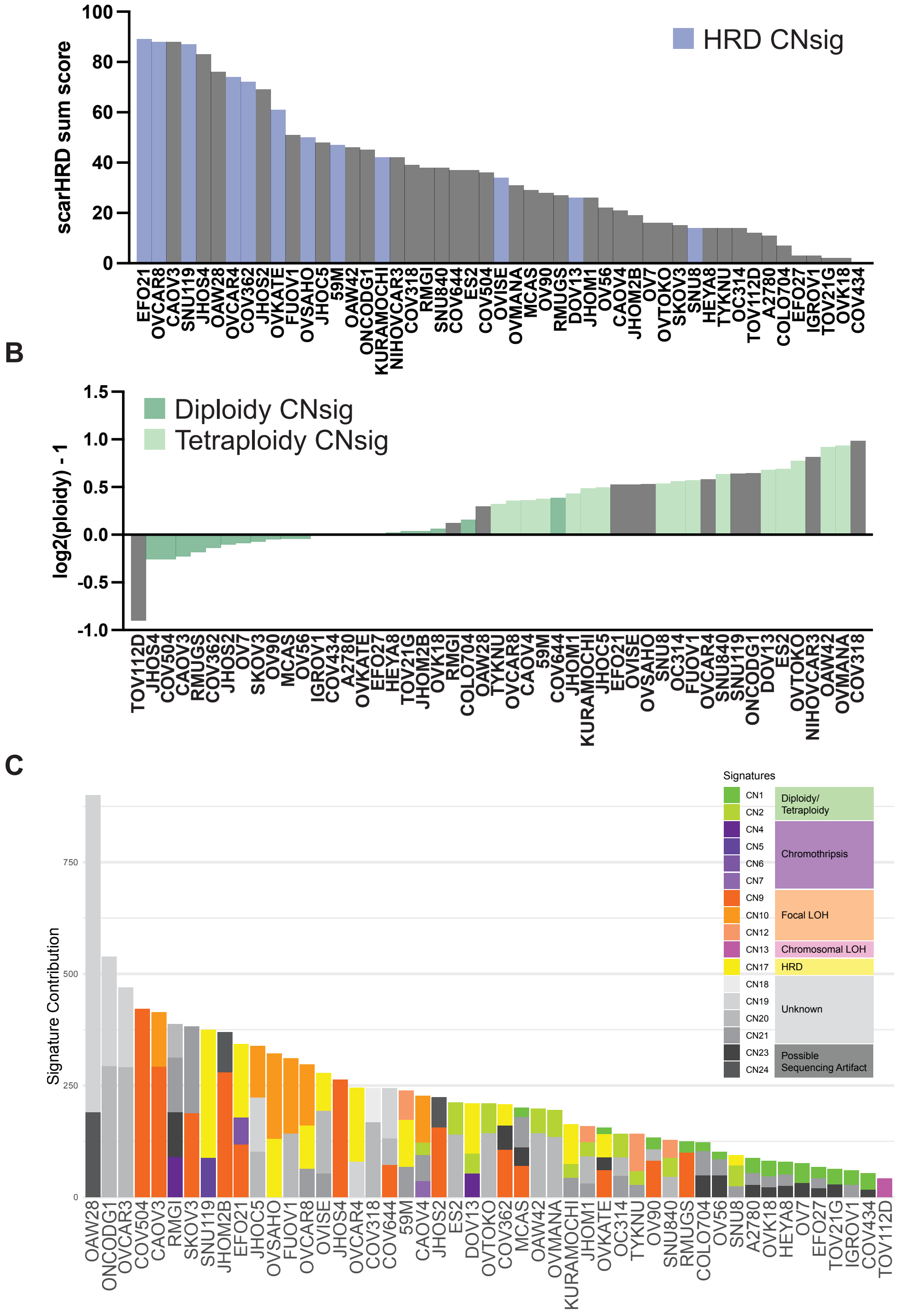

Supplemental Figure 3

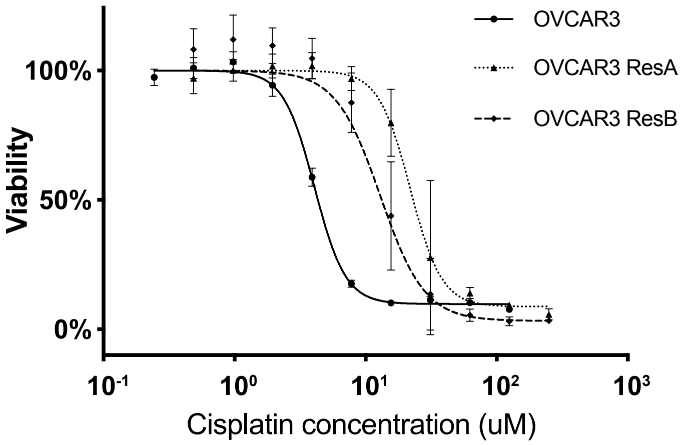

	OVCAR3	OVCAR3 ResA	OVCAR3 ResB
IC50	4.081	21.91	12.95

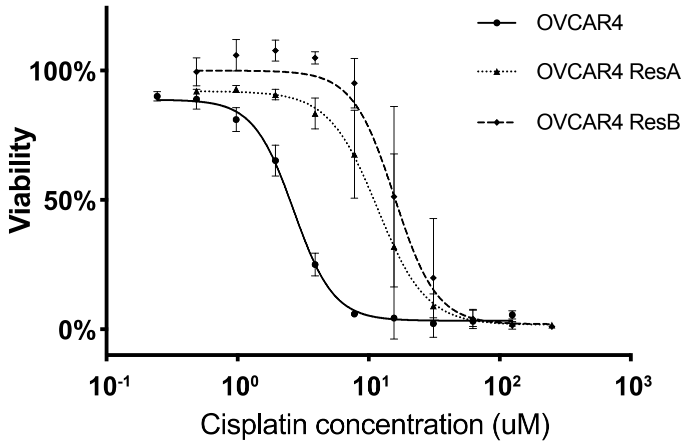

	OVCAR4	OVCAR4 ResA	OVCAR4 ResB
IC50	2.682	11.68	15.81

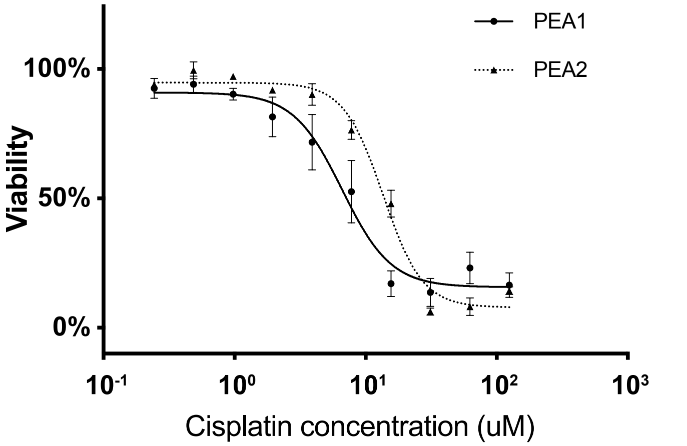

	PEA1	PEA2
IC50	6.640	13.62

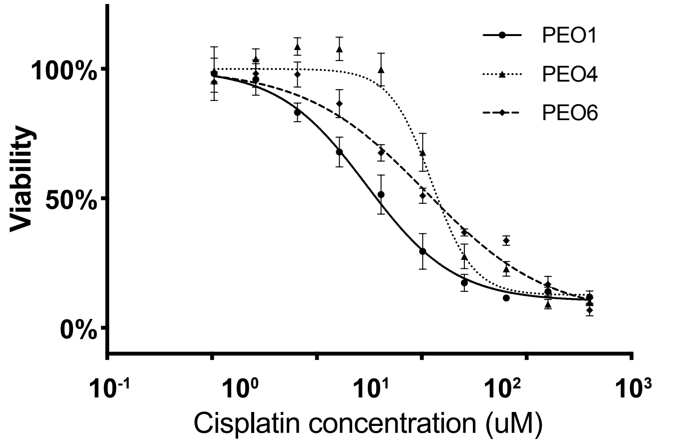

	PEO1	PEO4	PEO6
IC50	2.972	12.45	11.07

Supplemental Figure 4 - OVCAR3 ResA (1 of 2)

Enhanced repair and tolerance of platinum induced DNA damage and blockage of cell cycle inhibition

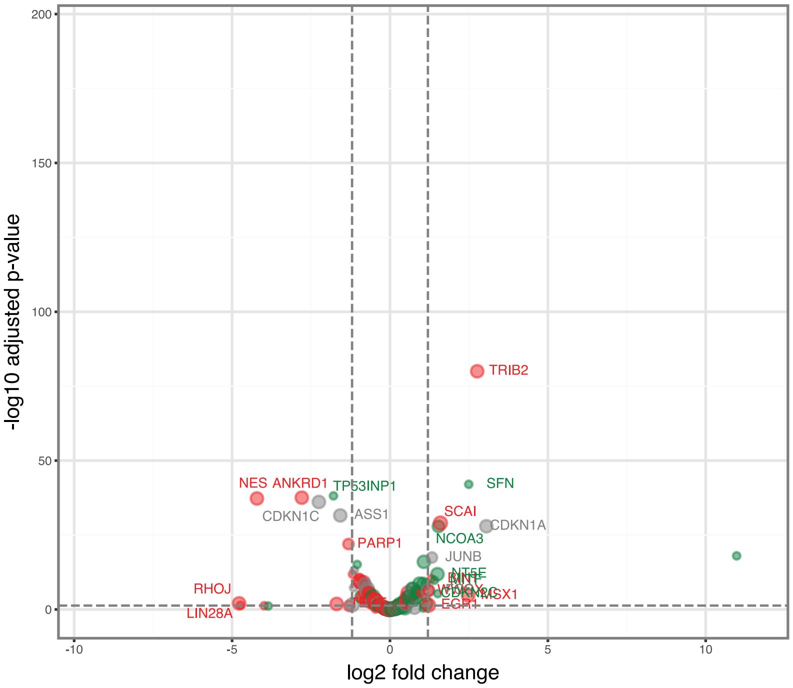

Extracellular mechanisms that alter the extracellular matrix (ECM) and enhance tumor-promoting inflammation

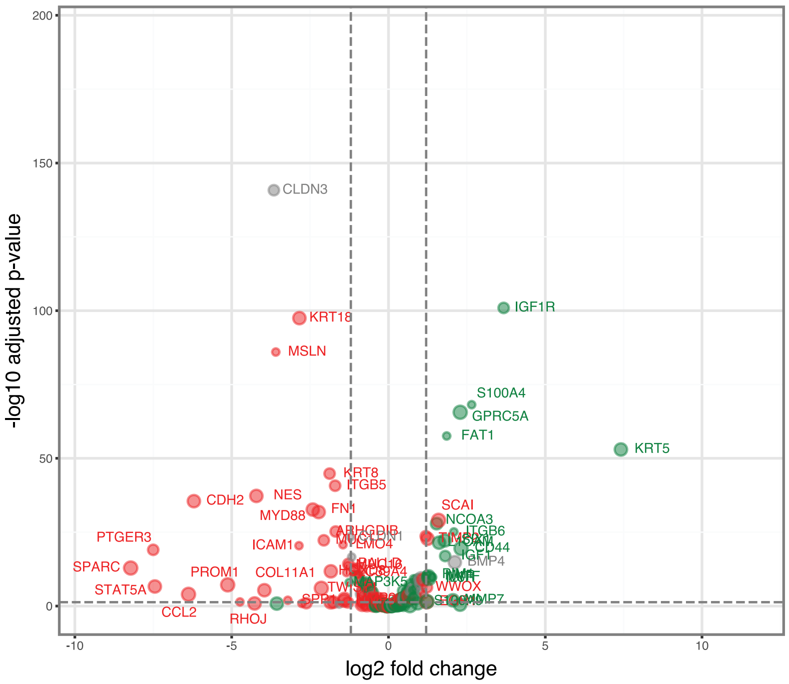

Hypoxia and other stress responses (e.g. ER stress response)

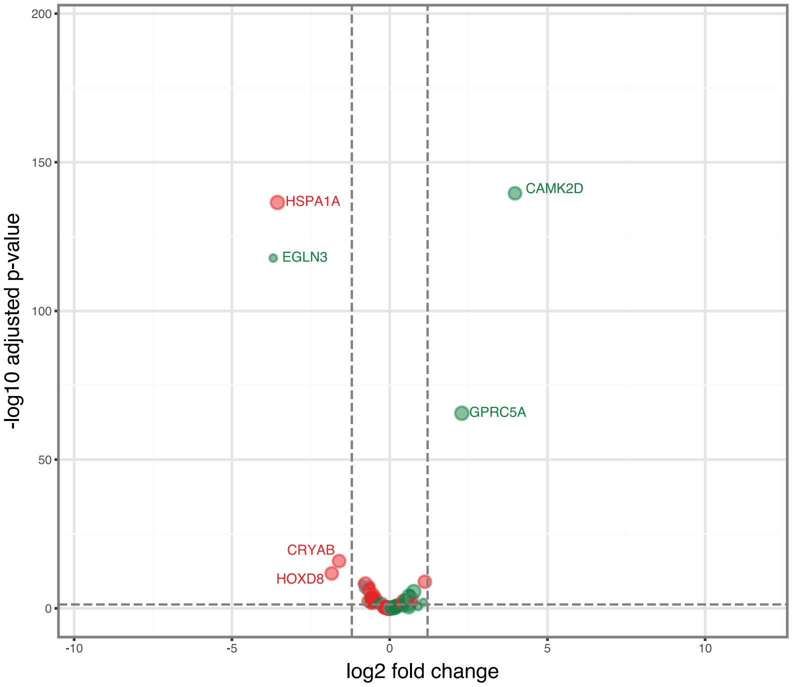

Inhibition of apoptotic signaling, downregulation of reactive oxygen species (ROS), and increased autophagy

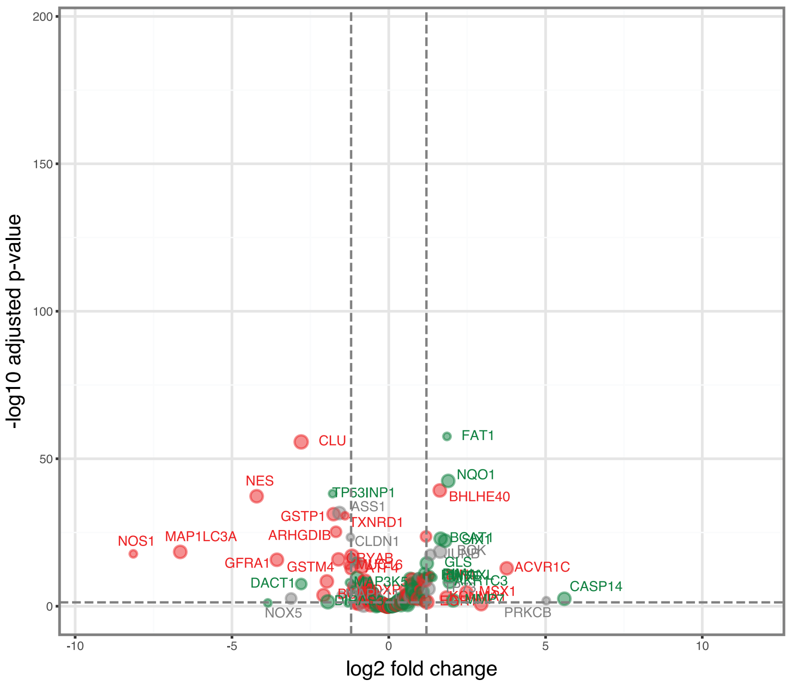

Supplemental Figure 4 - OVCAR3 ResA (2 of 2)

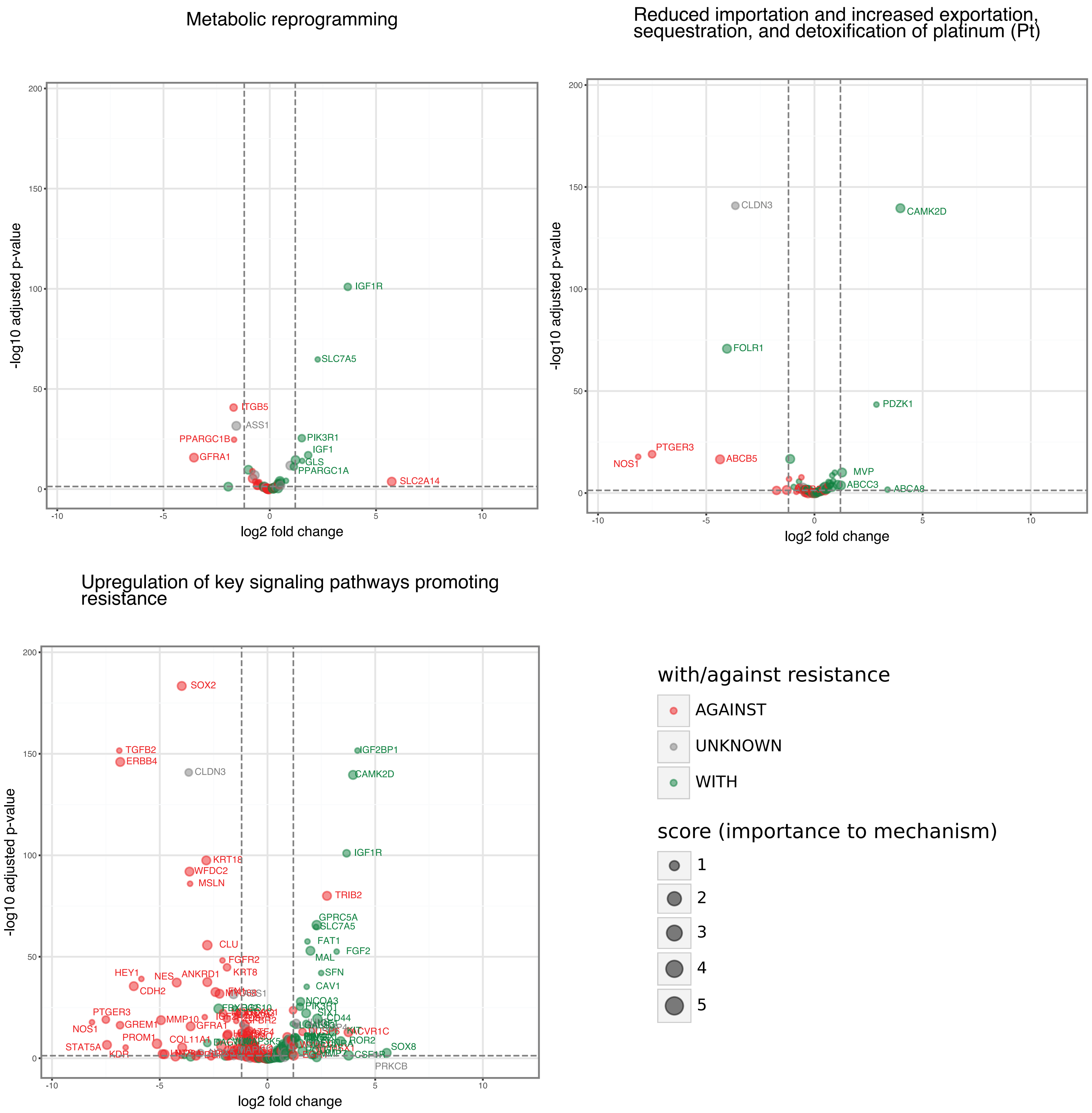

Supplemental Figure 4 - OVCAR3 ResB (1 of 2)

Enhanced repair and tolerance of platinum induced DNA damage and blockage of cell cycle inhibition

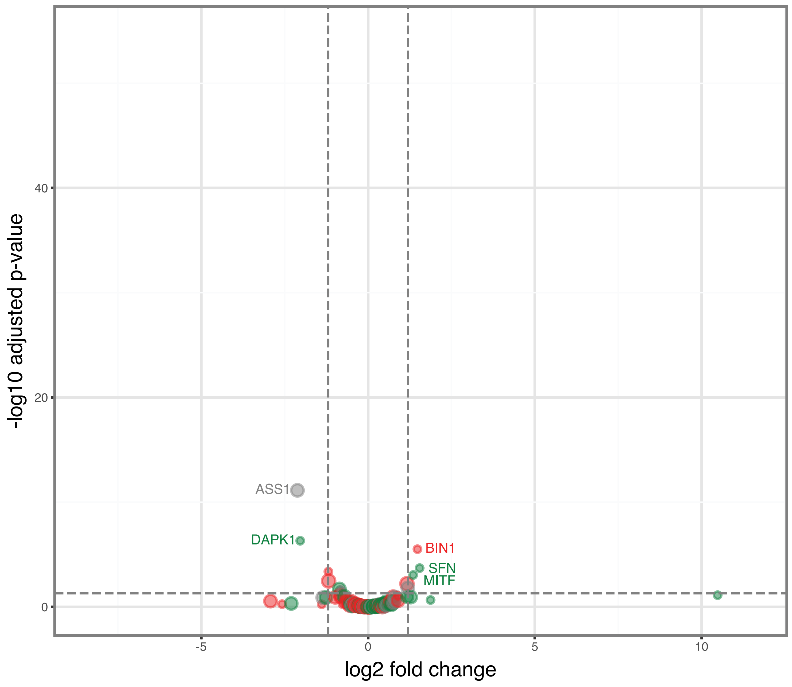

Extracellular mechanisms that alter the extracellular matrix (ECM) and enhance tumor-promoting inflammation

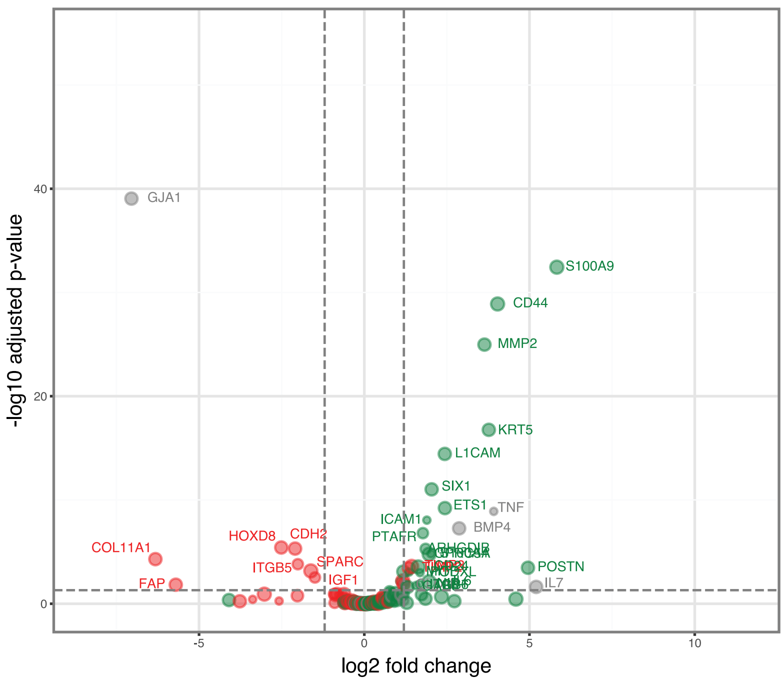

Hypoxia and other stress responses (e.g. ER stress response)

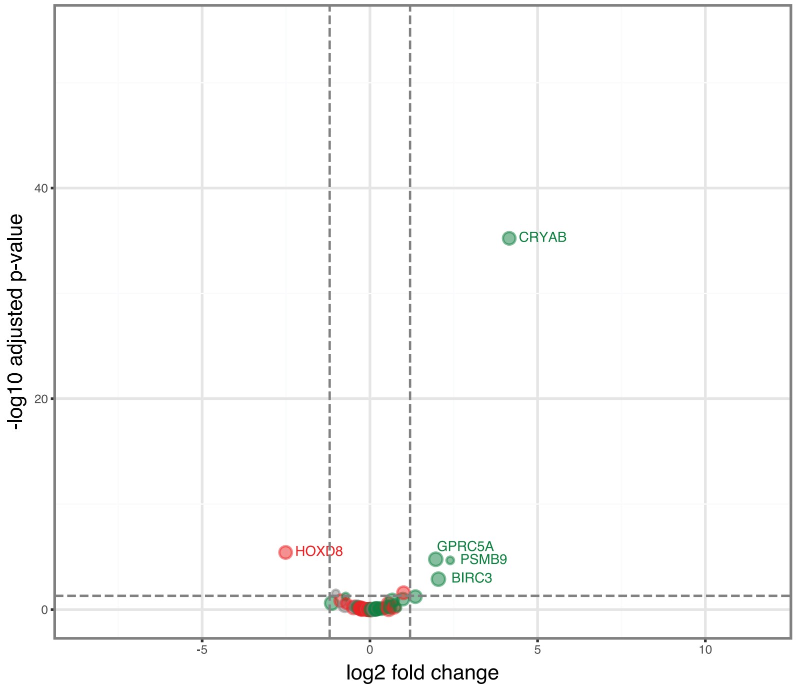

Inhibition of apoptotic signaling, downregulation of reactive oxygen species (ROS), and increased autophagy

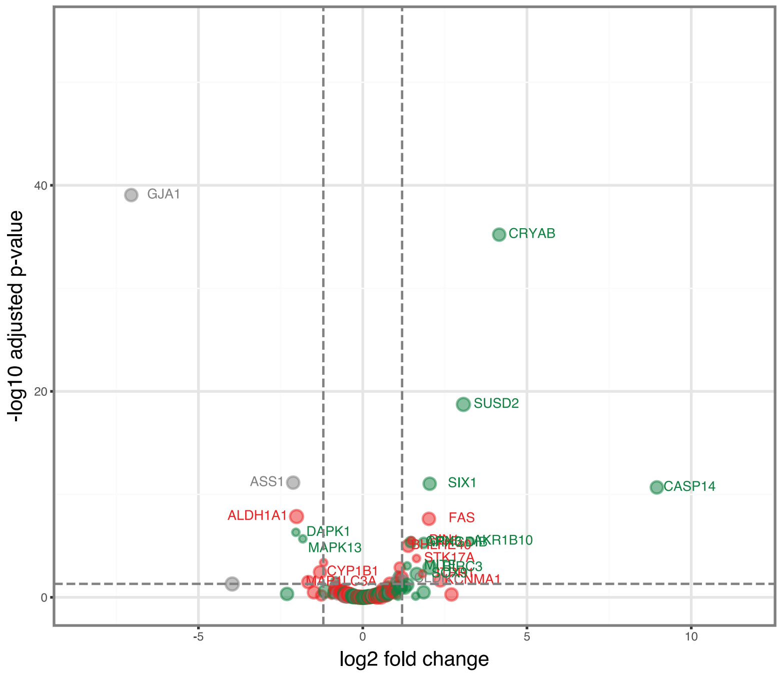

Supplemental Figure 4 - OVCAR3 ResB (2 of 2)

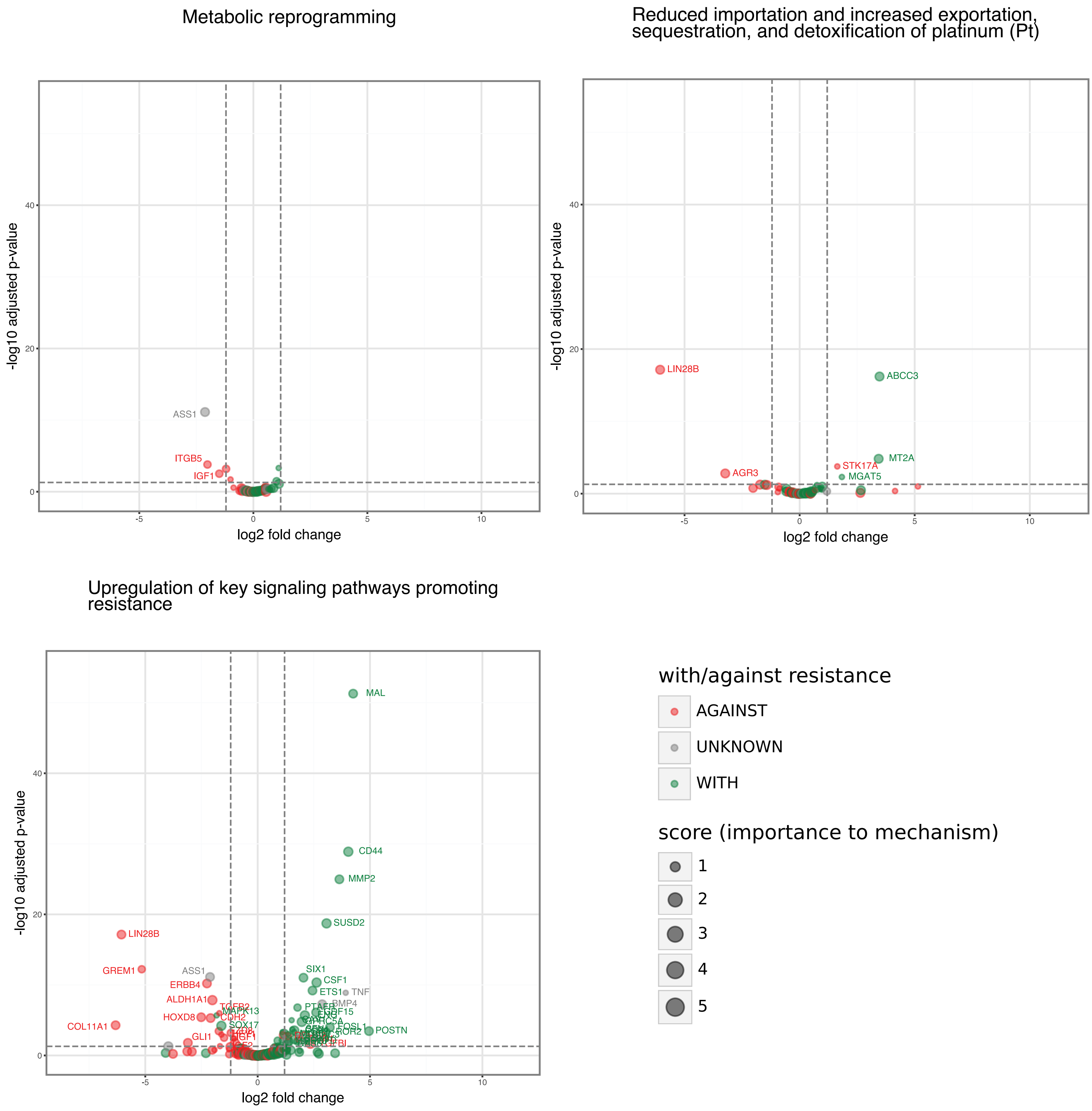

Supplemental Figure 4 - OVCAR4 ResA (1 of 2)

Enhanced repair and tolerance of platinum induced DNA damage and blockage of cell cycle inhibition

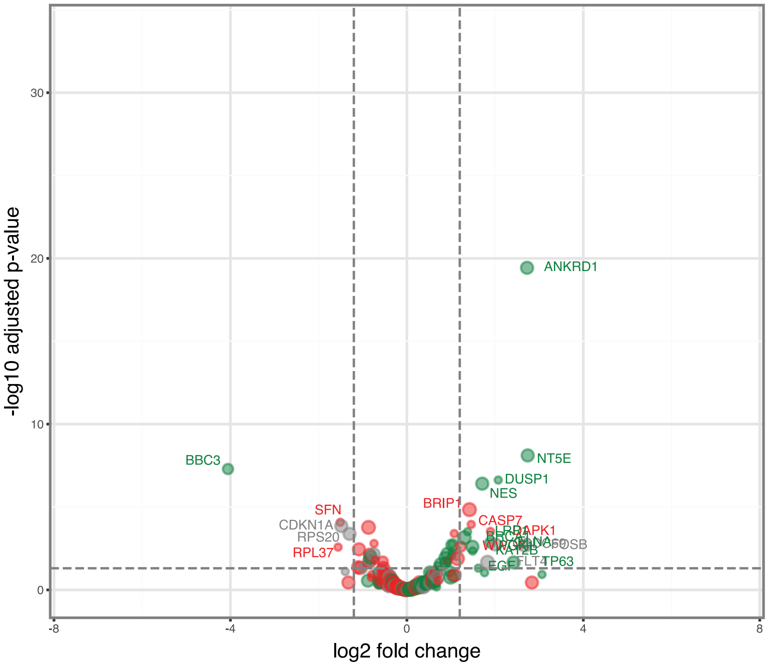

Extracellular mechanisms that alter the extracellular matrix (ECM) and enhance tumor-promoting inflammation

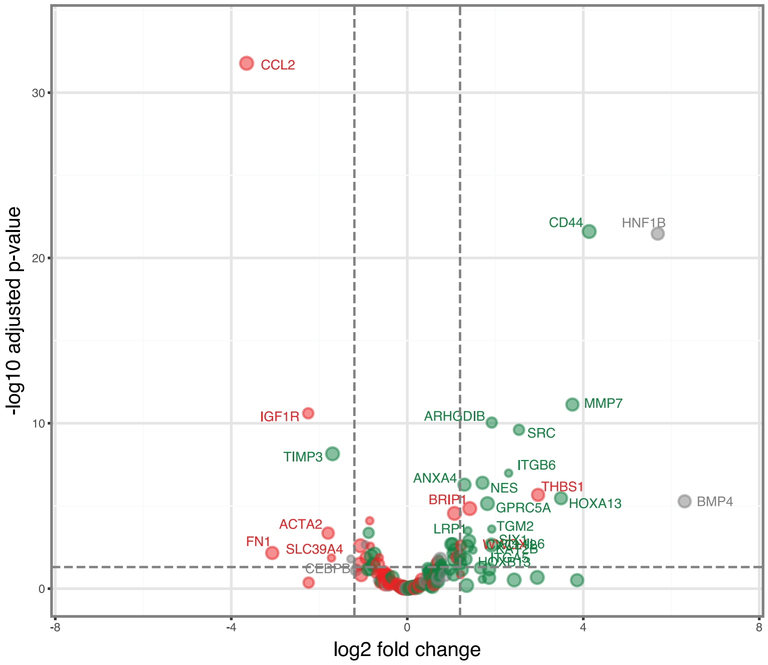

Hypoxia and other stress responses (e.g. ER stress response)

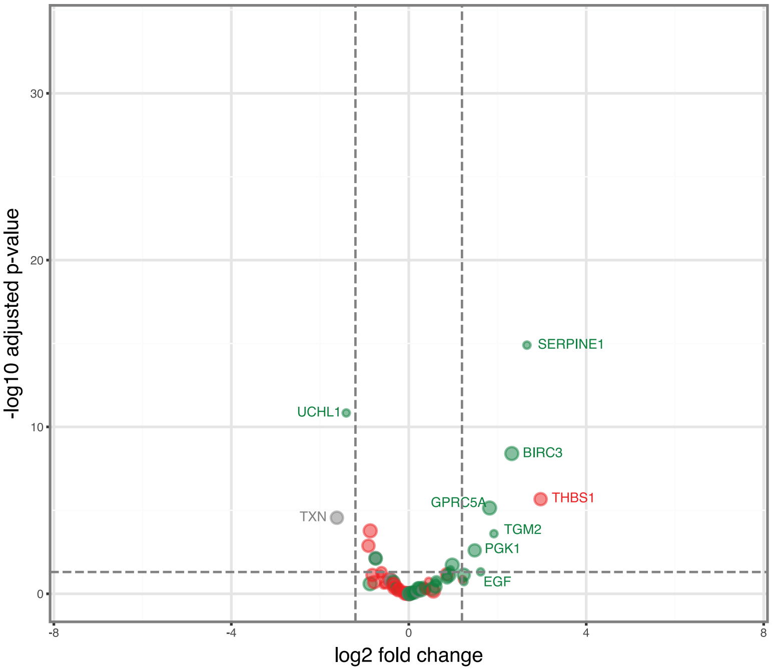

Inhibition of apoptotic signaling, downregulation of reactive oxygen species (ROS), and increased autophagy

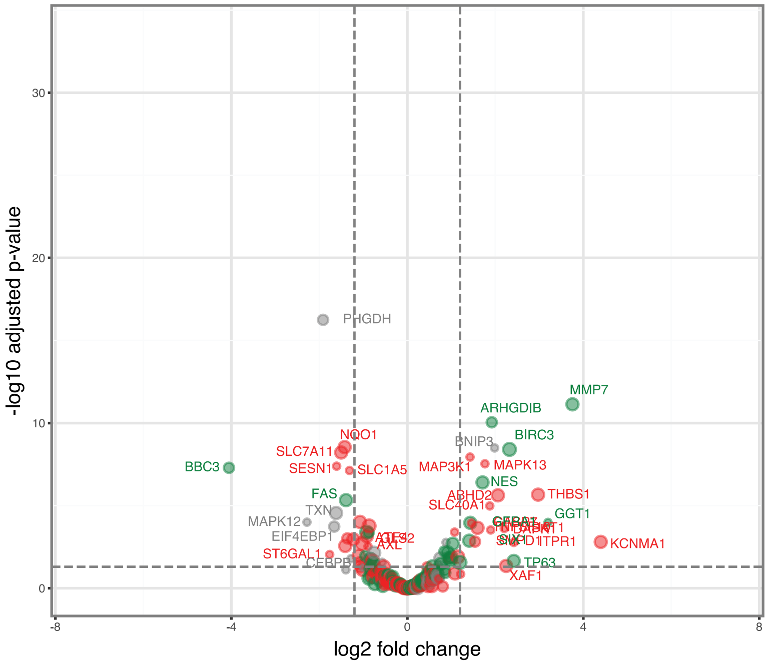

Supplemental Figure 4 - OVCAR4 ResA (2 of 2)

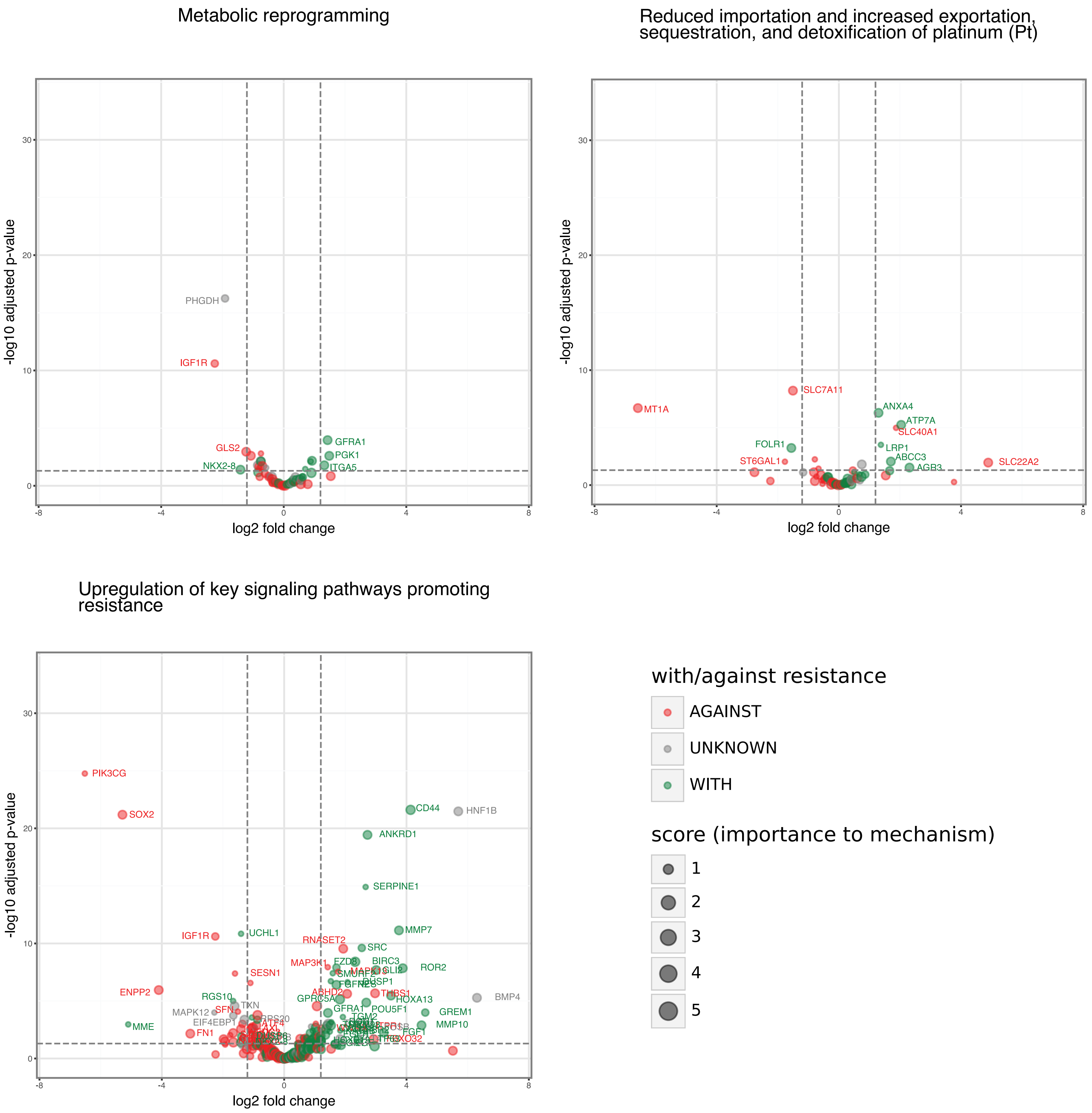

Supplemental Figure 4 - OVCAR4 ResB (1 of 2)

Enhanced repair and tolerance of platinum induced DNA damage and blockage of cell cycle inhibition

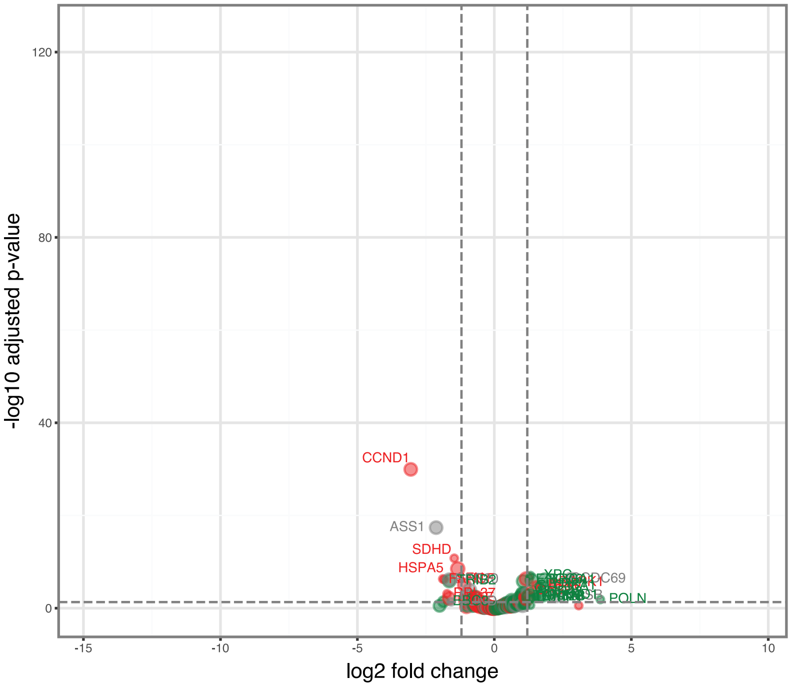

Extracellular mechanisms that alter the extracellular matrix (ECM) and enhance tumor-promoting inflammation

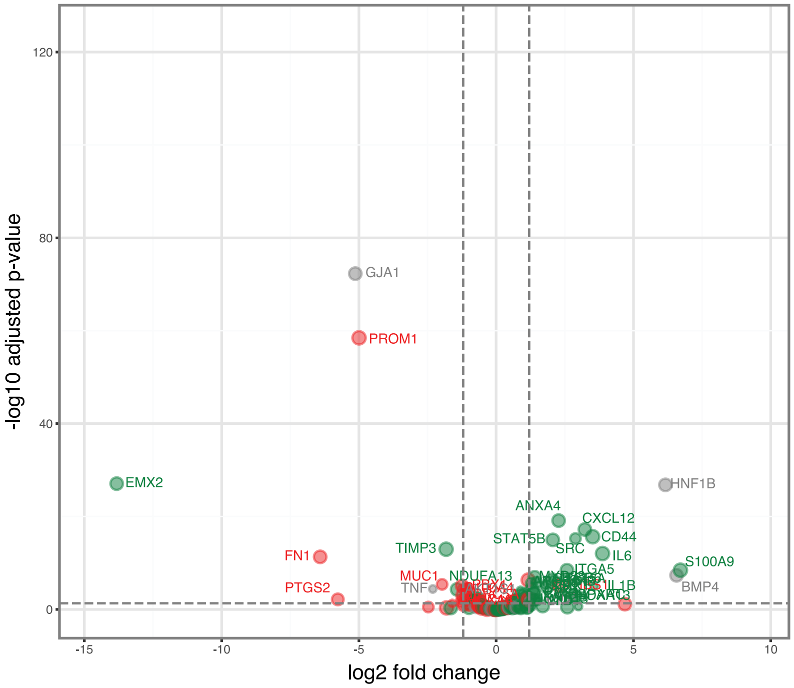

Hypoxia and other stress responses (e.g. ER stress response)

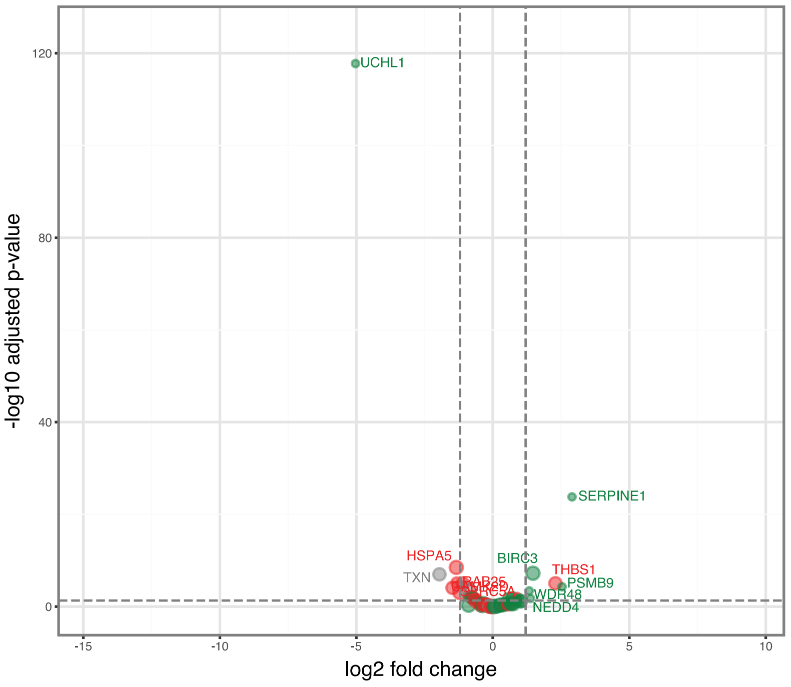

Inhibition of apoptotic signaling, downregulation of reactive oxygen species (ROS), and increased autophagy

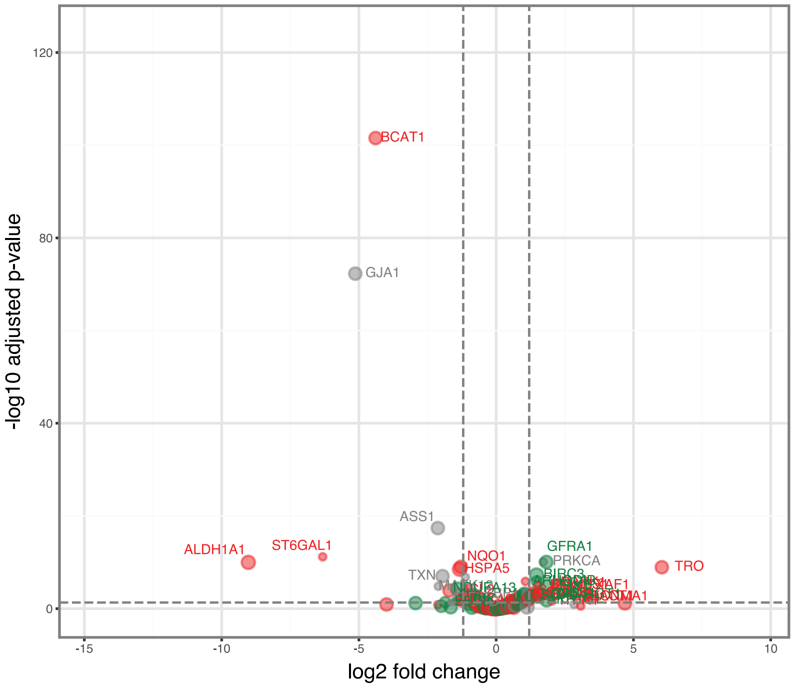

Supplemental Figure 4 - OVCAR4 ResB (2 of 2)

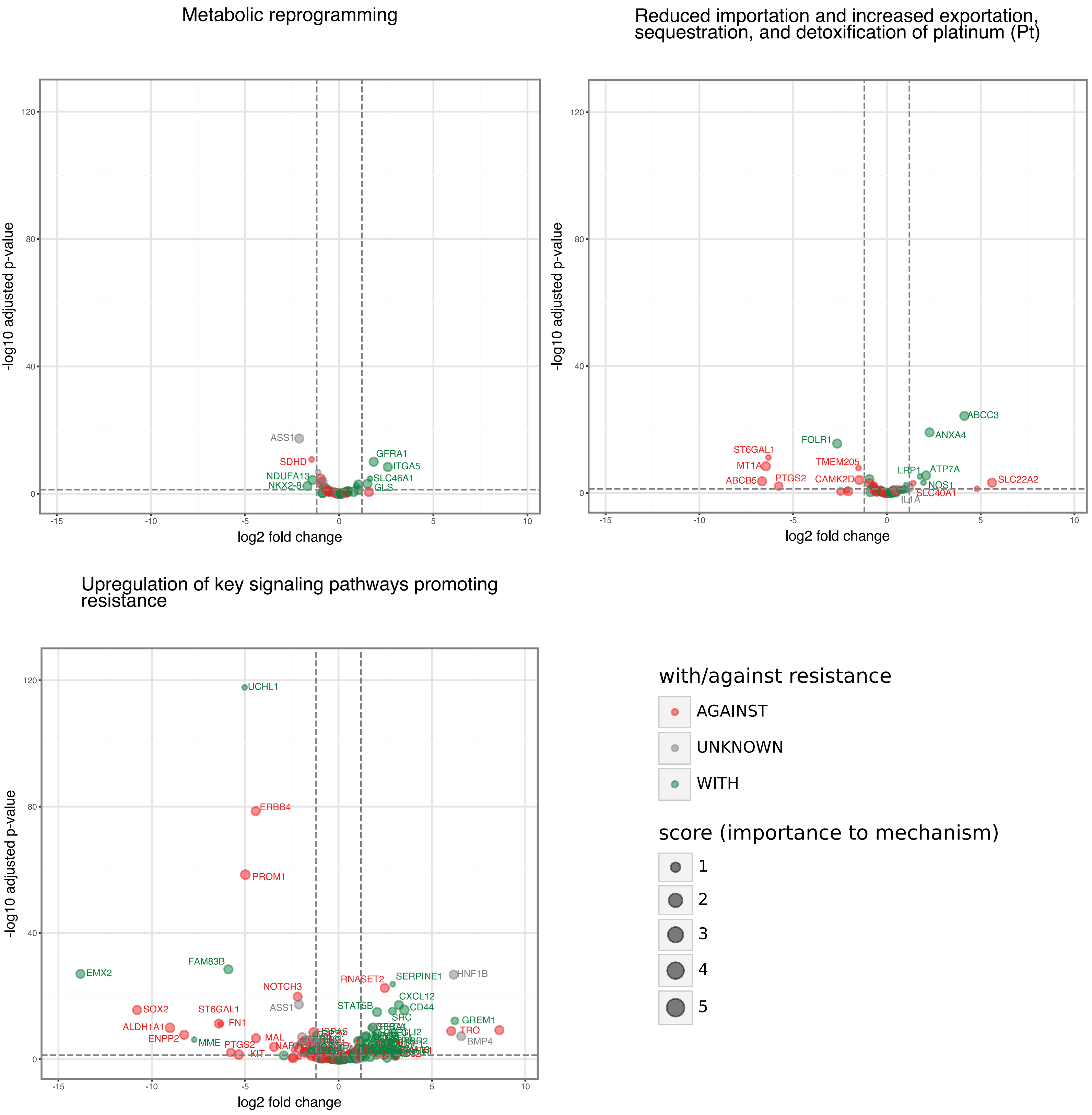

Supplemental Figure 4 - PEO4 (1 of 2)

Enhanced repair and tolerance of platinum induced DNA damage and blockage of cell cycle inhibition

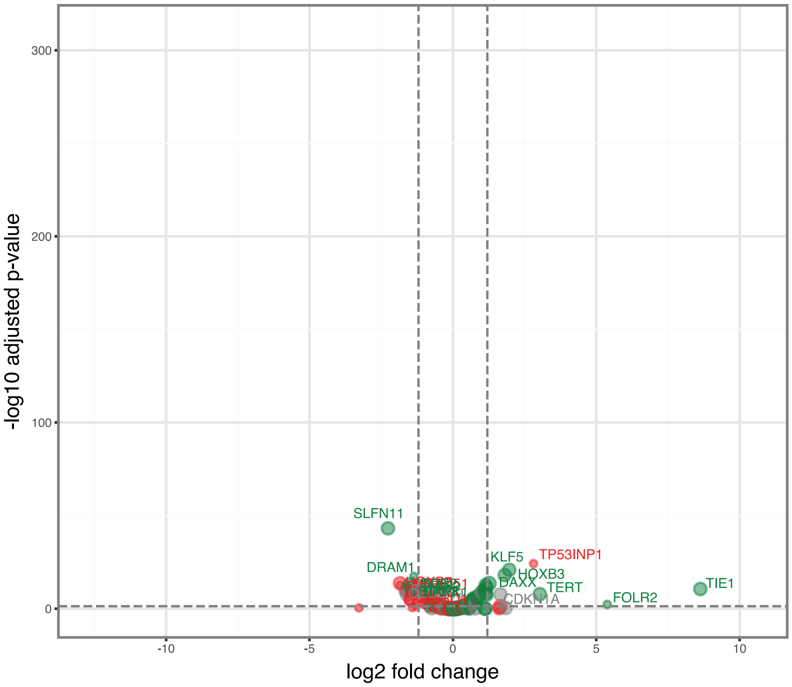

Extracellular mechanisms that alter the extracellular matrix (ECM) and enhance tumor-promoting inflammation

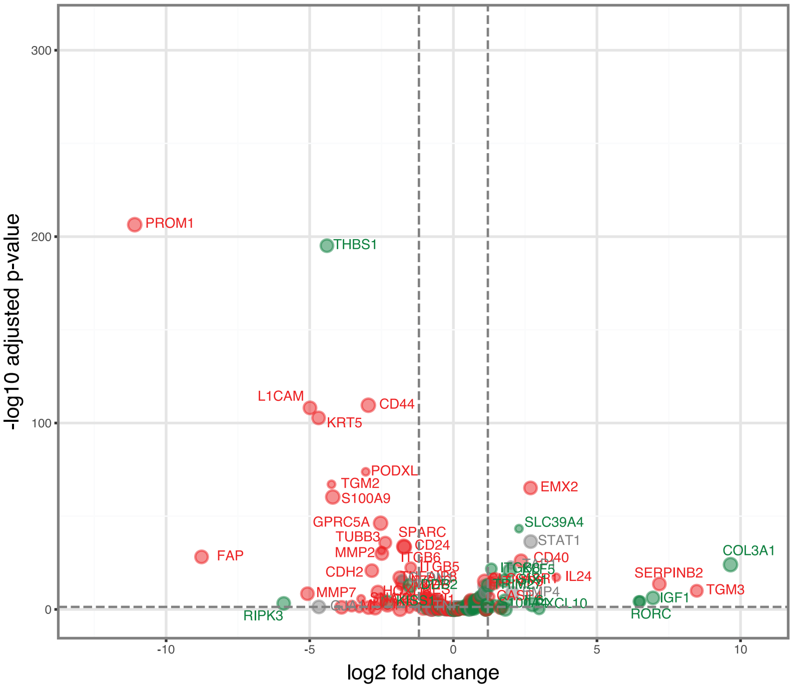

Hypoxia and other stress responses (e.g. ER stress response)

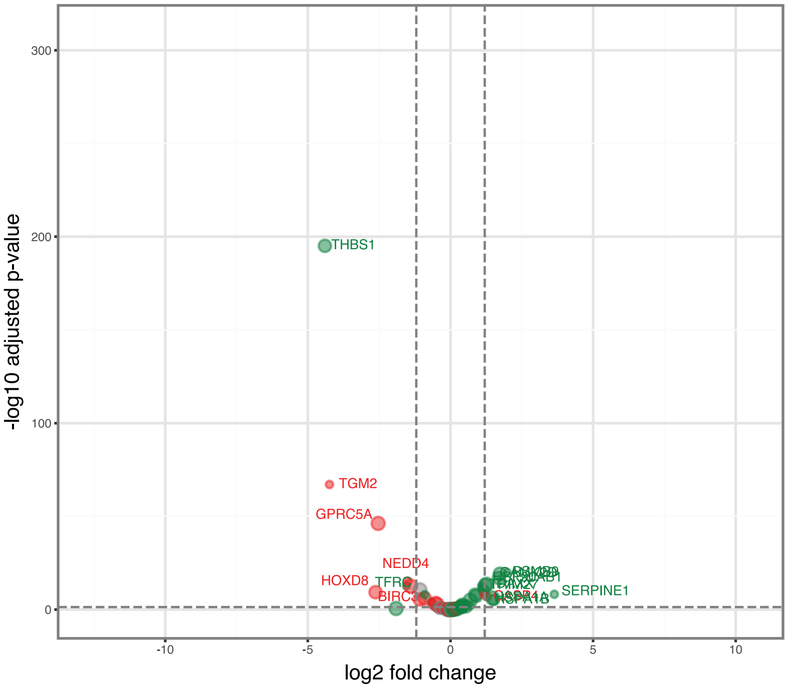

Inhibition of apoptotic signaling, downregulation of reactive oxygen species (ROS), and increased autophagy

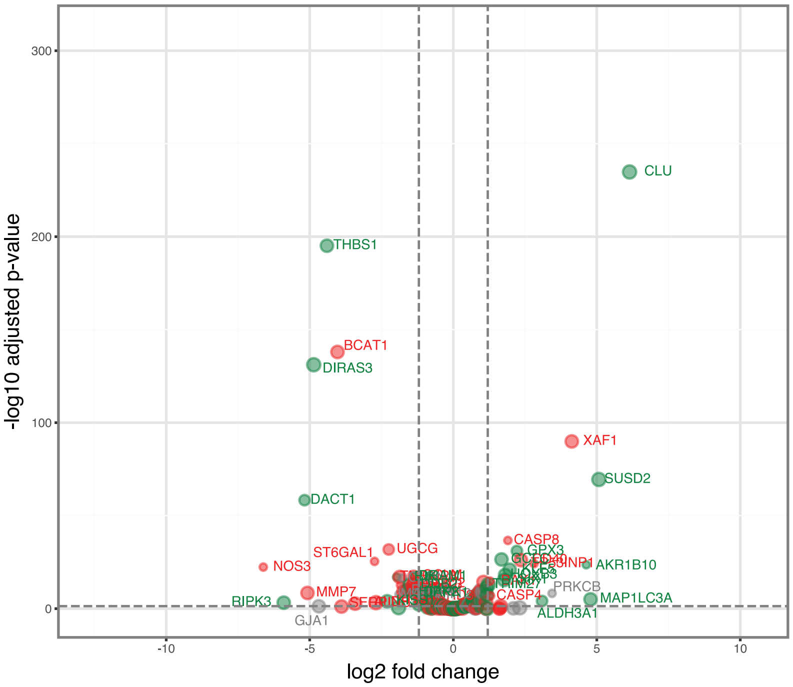

Supplemental Figure 4 - PEO4 (2 of 2)

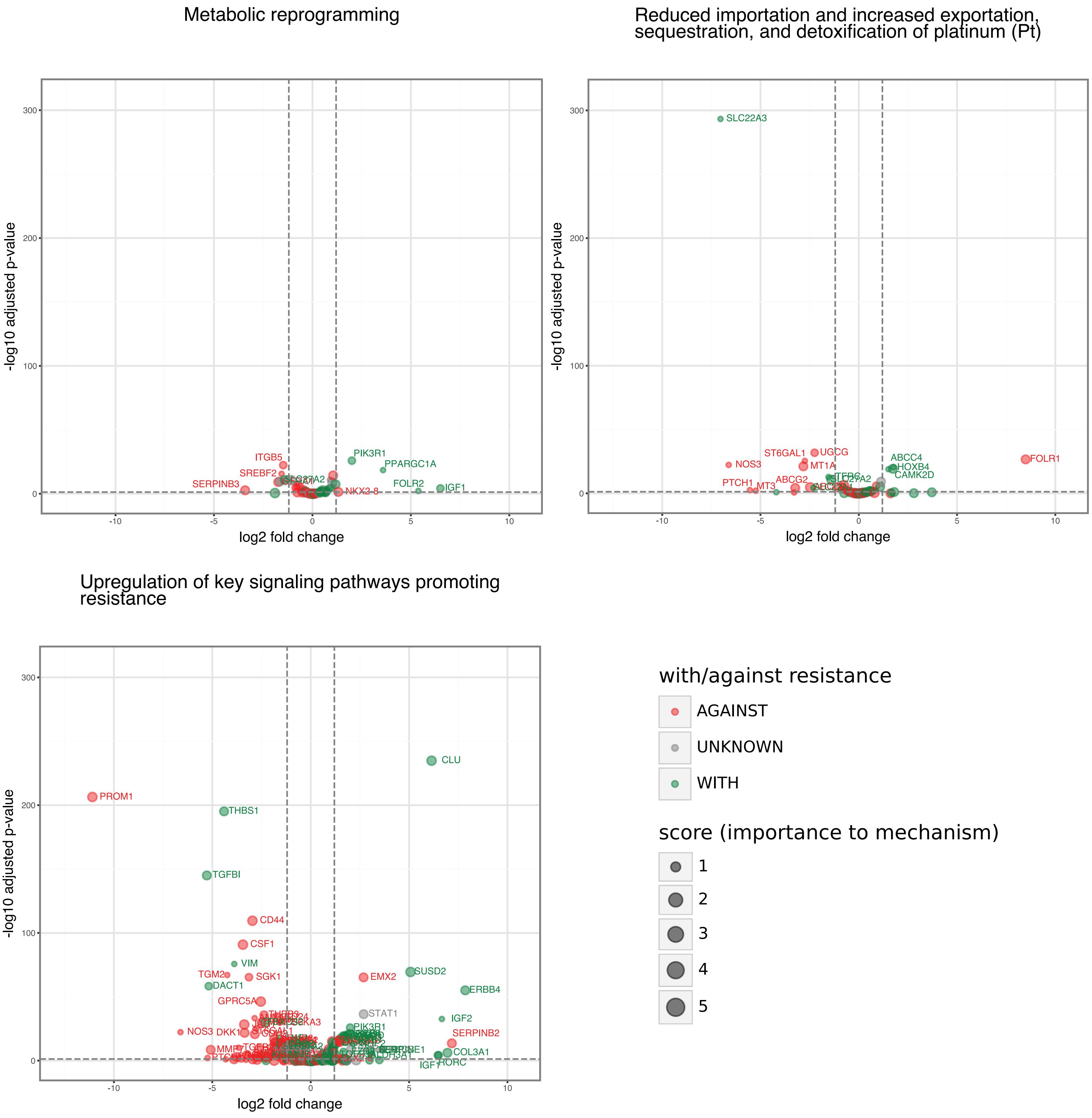

Supplemental Figure 4 - PEO6 (1 of 2)

Enhanced repair and tolerance of platinum induced DNA damage and blockage of cell cycle inhibition

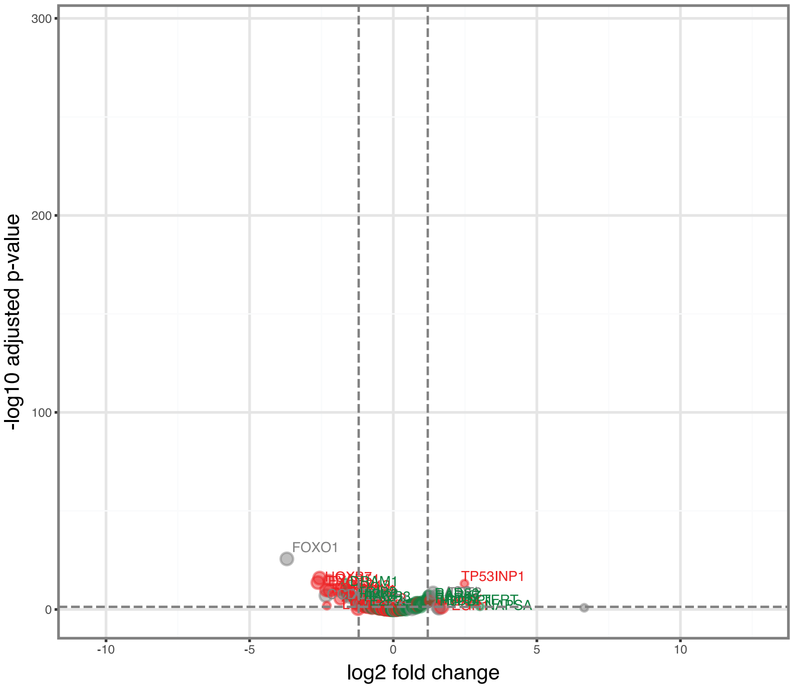

Extracellular mechanisms that alter the extracellular matrix (ECM) and enhance tumor-promoting inflammation

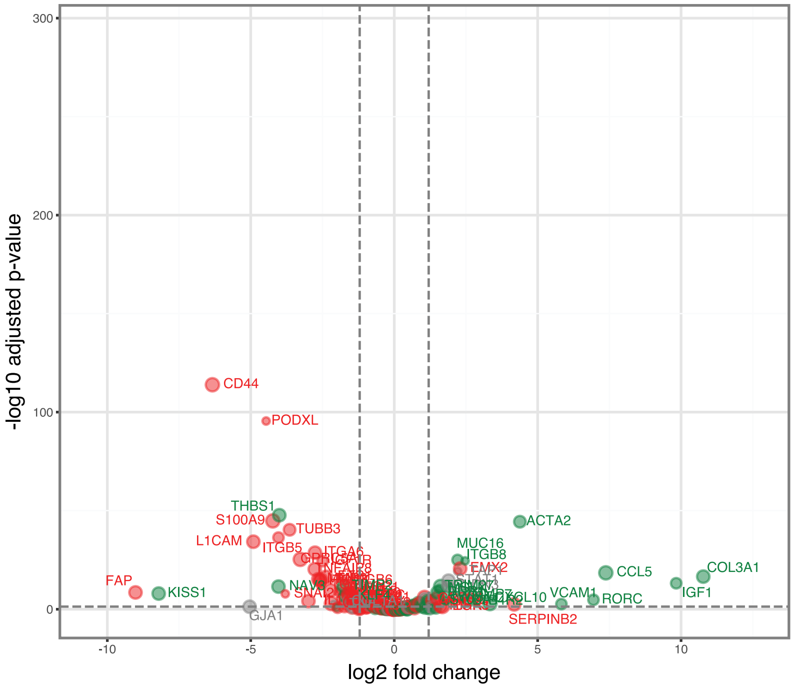

Hypoxia and other stress responses (e.g. ER stress response)

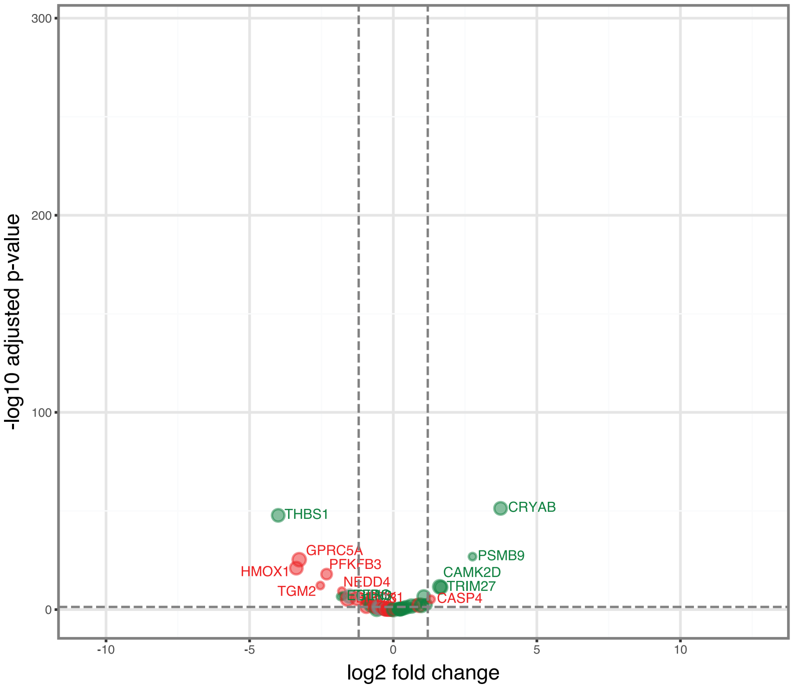

Inhibition of apoptotic signaling, downregulation of reactive oxygen species (ROS), and increased autophagy

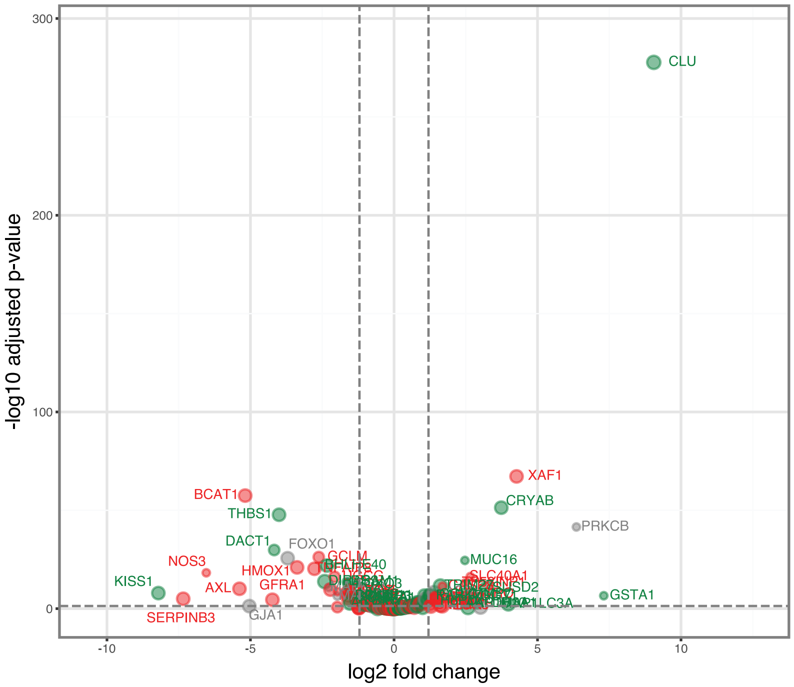

Supplemental Figure 4 - PEO6 (2 of 2)

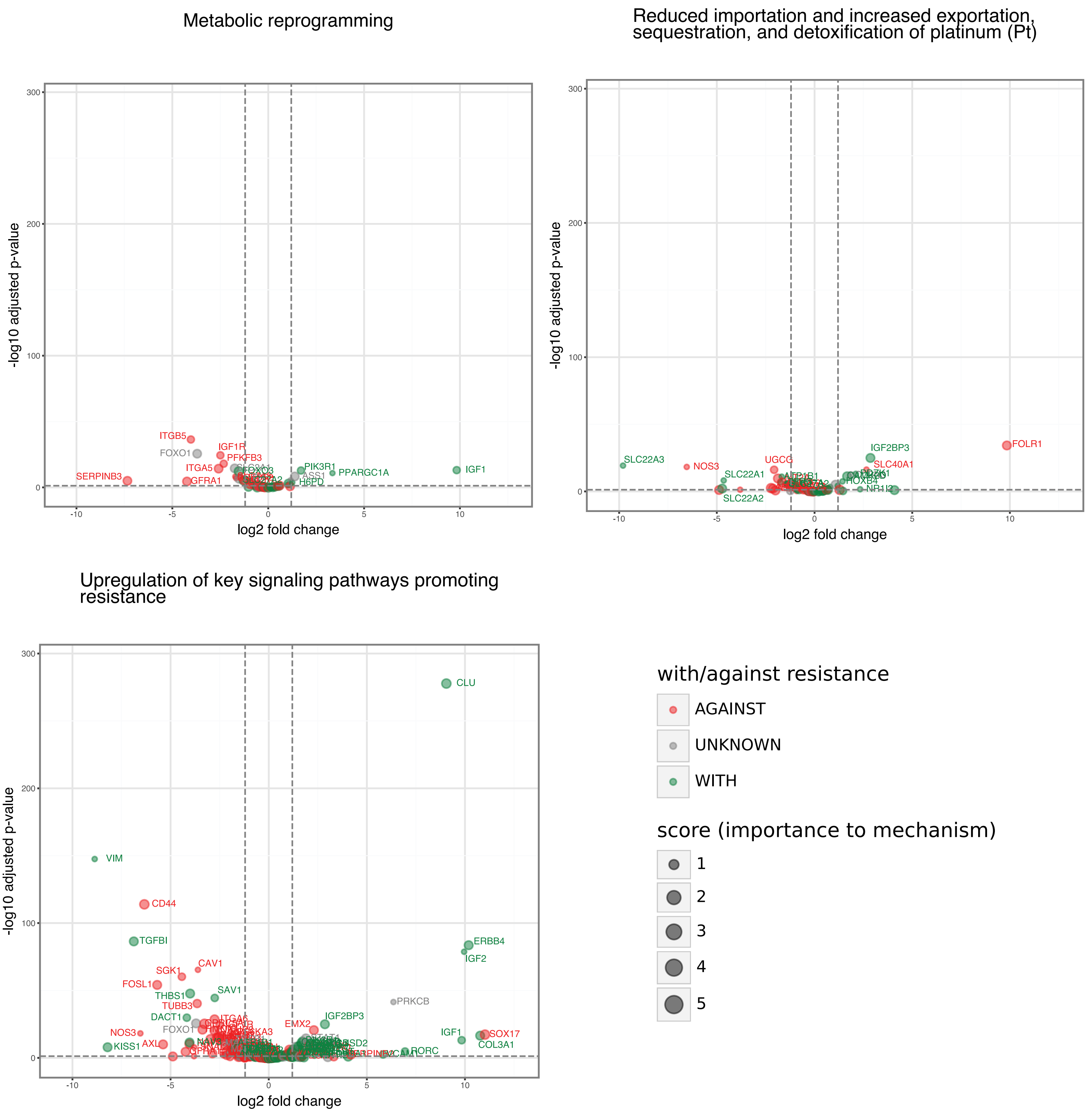

Supplemental Figure 4 - PEA2 (1 of 2)

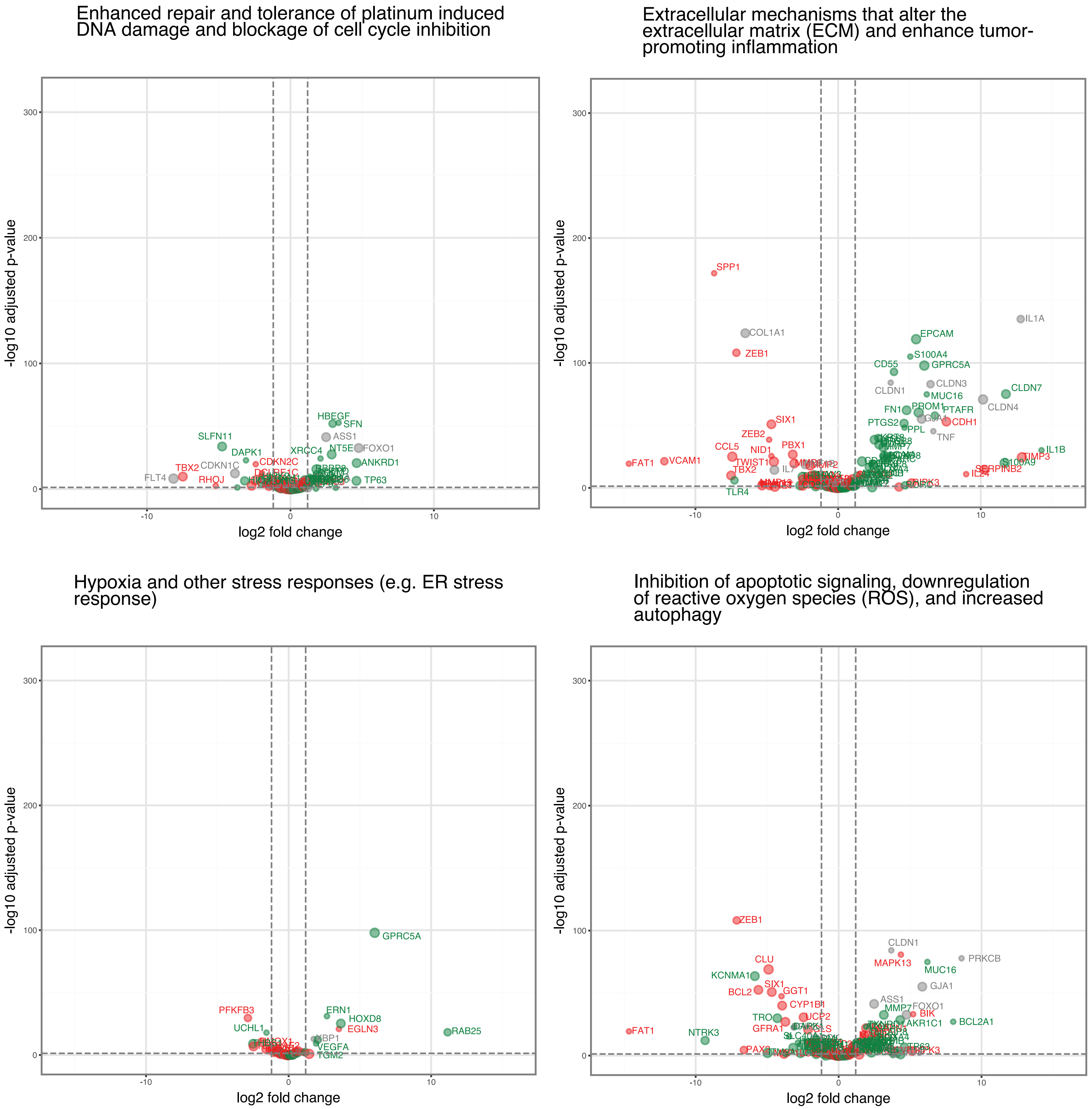

Supplemental Figure 4 - PEA2 (2 of 2)

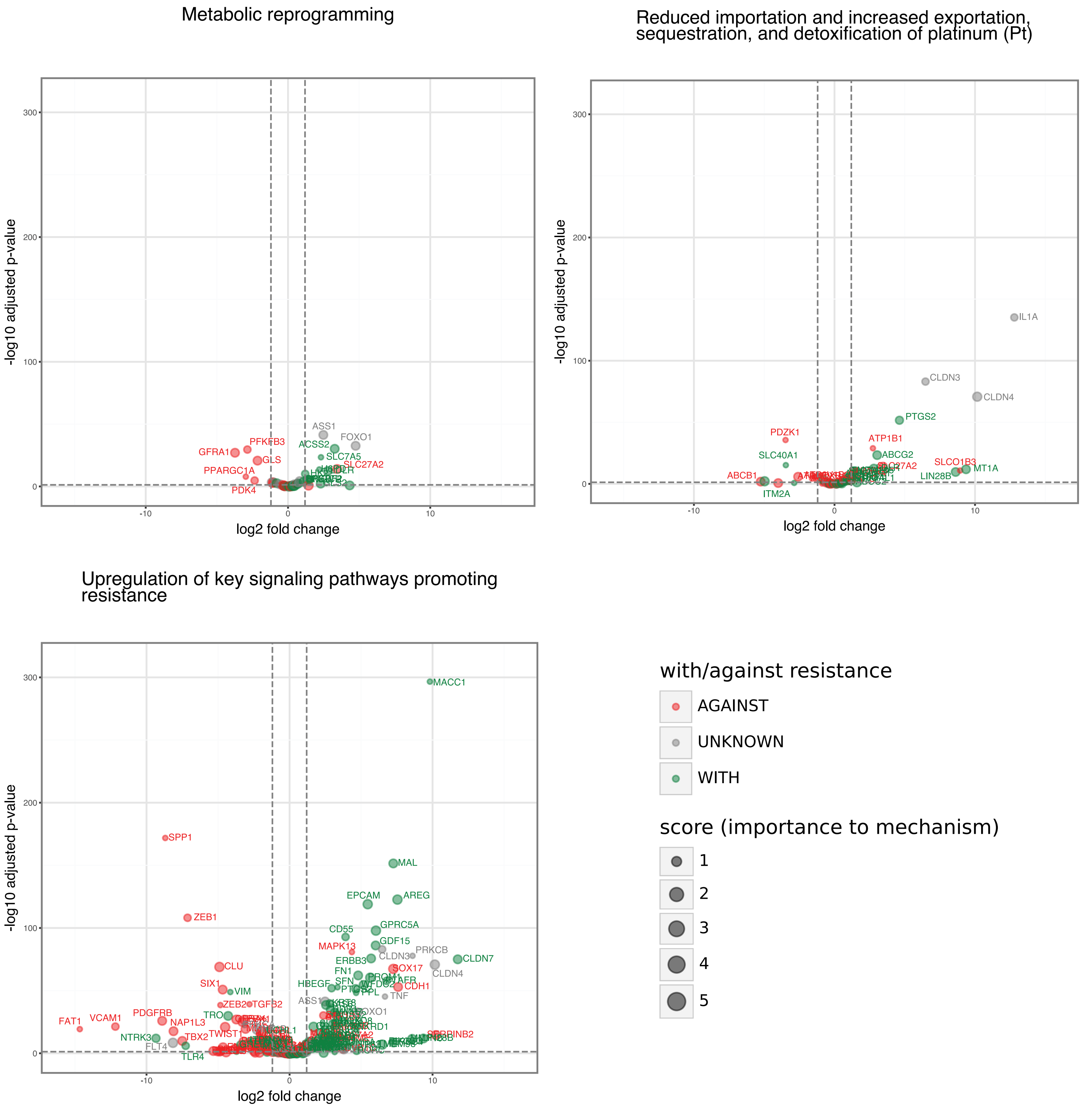

Supplemental Figure 5

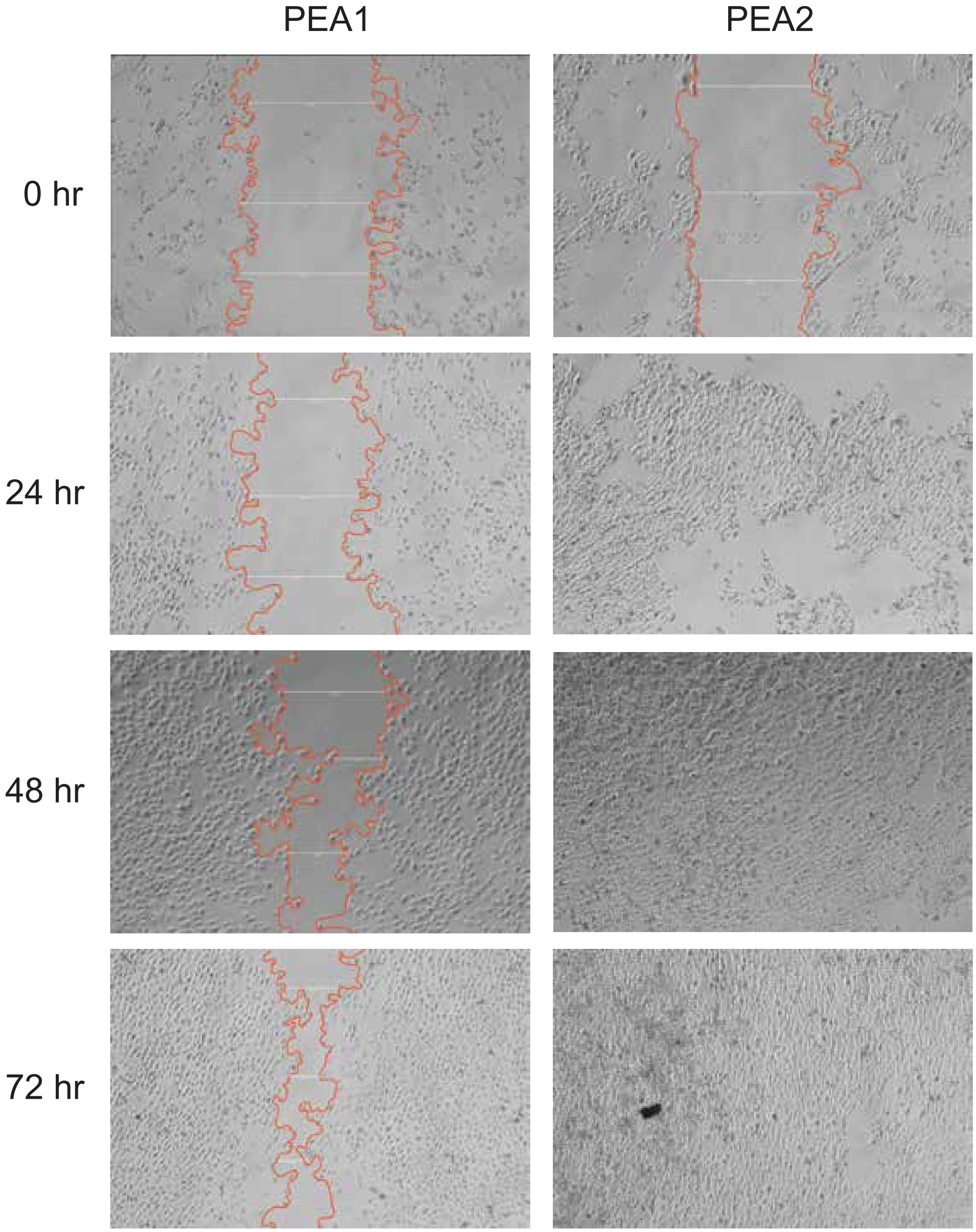

Supplemental Figure 6A - OVCAR3 Isogenic Lines

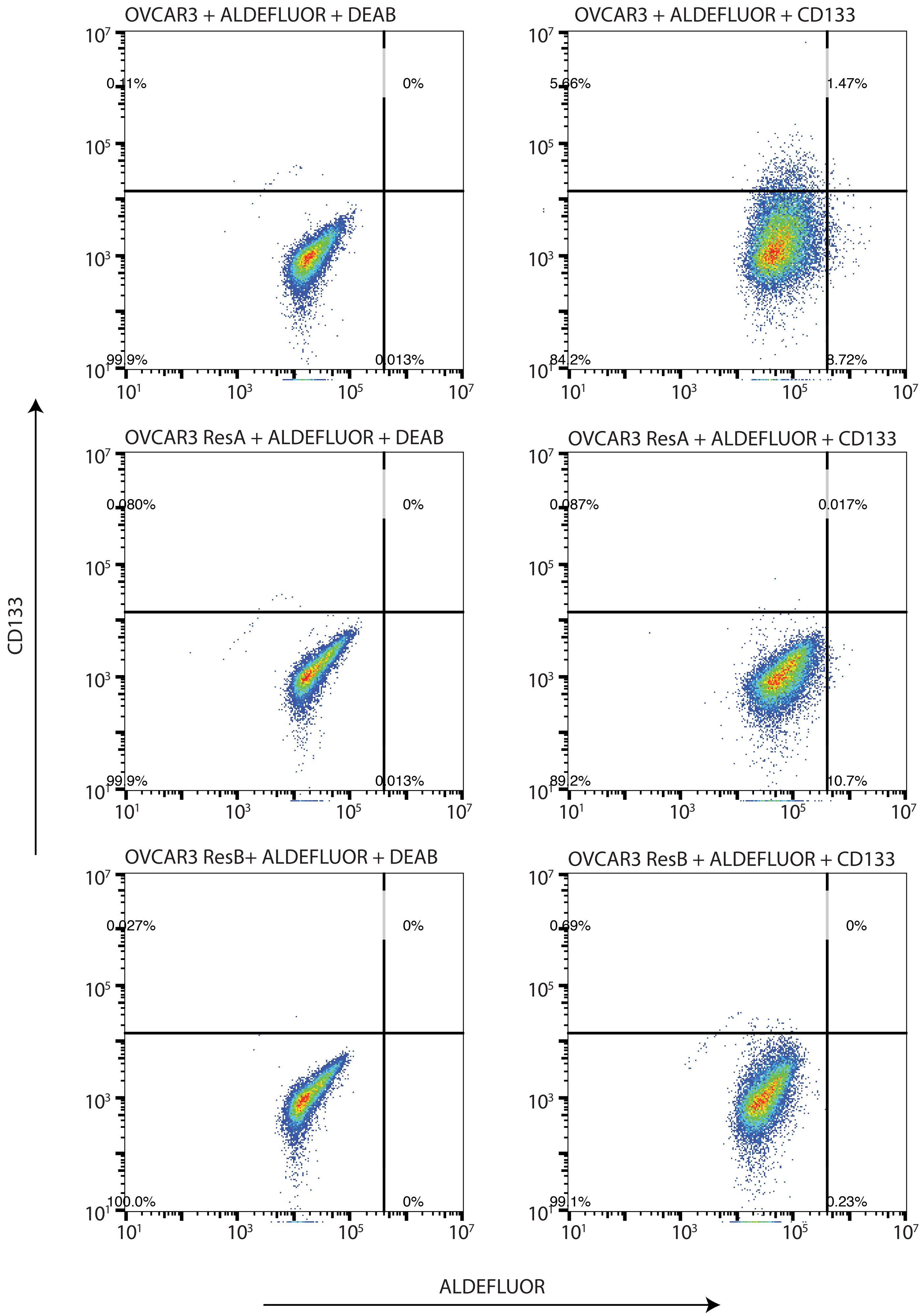

Supplemental Figure 6B - OVCAR4 Isogenic Lines

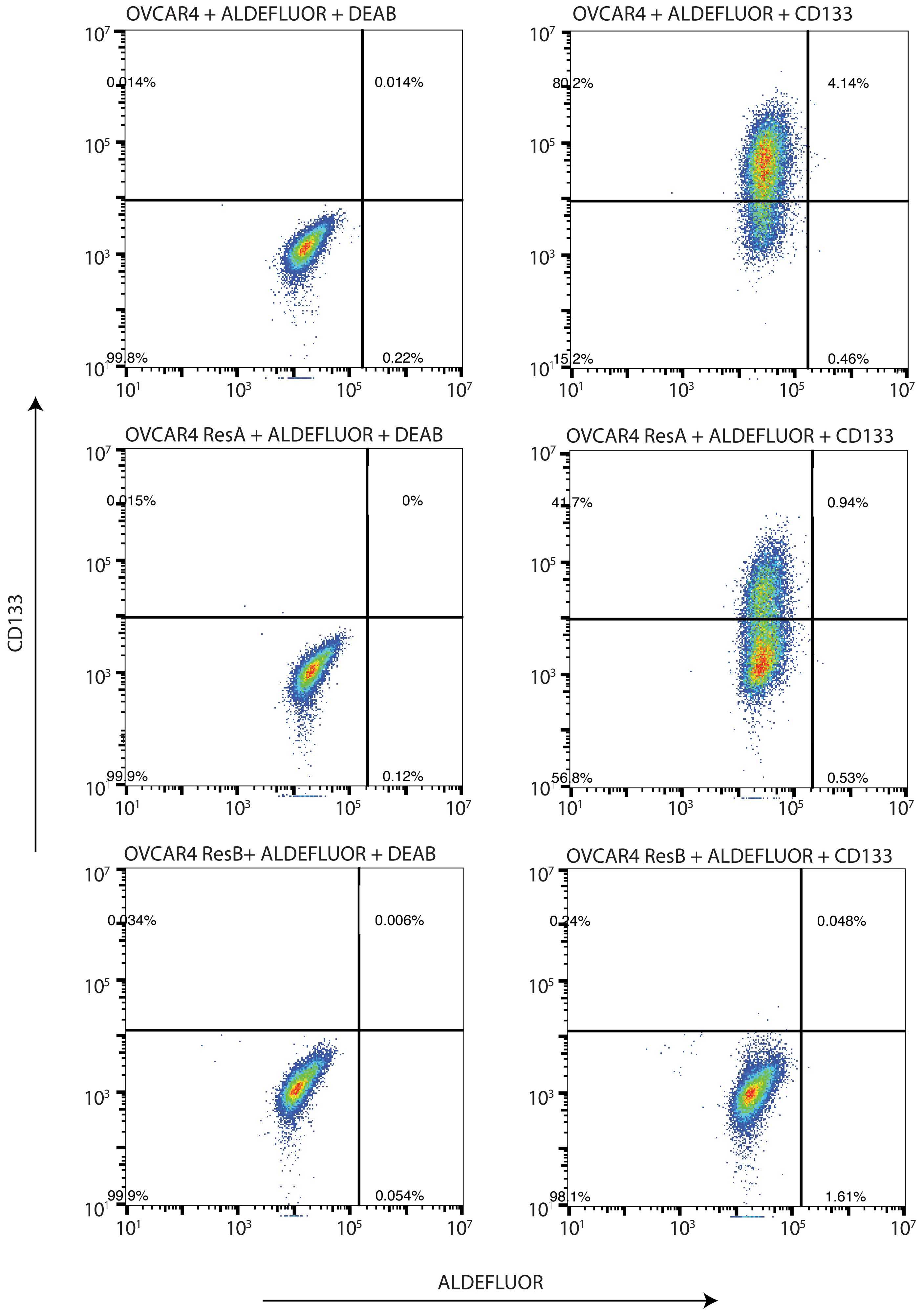

Supplemental Figure 6C - PEA Isogenic Lines

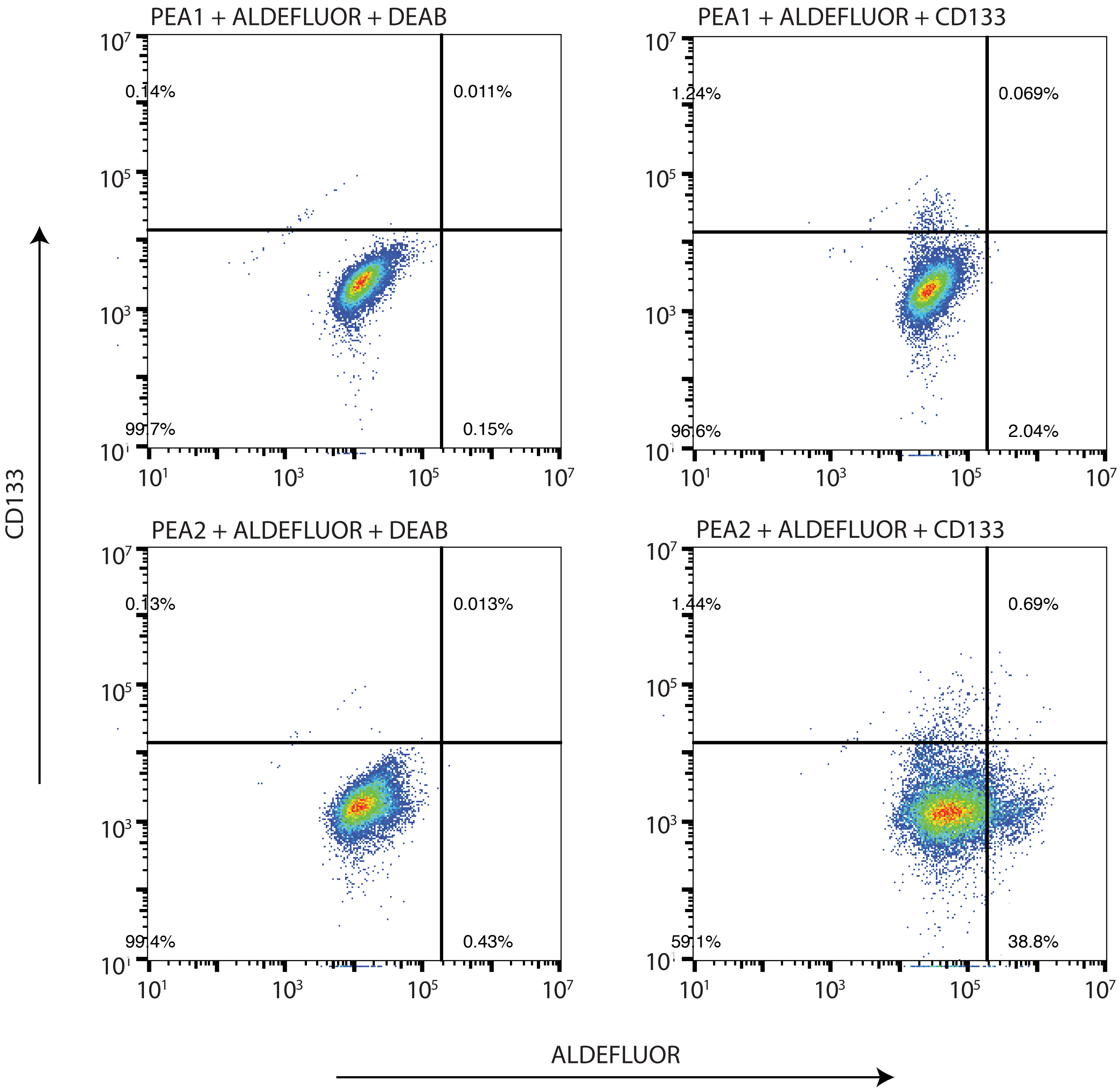

Supplemental Figure 6D - PEO Isogenic Lines

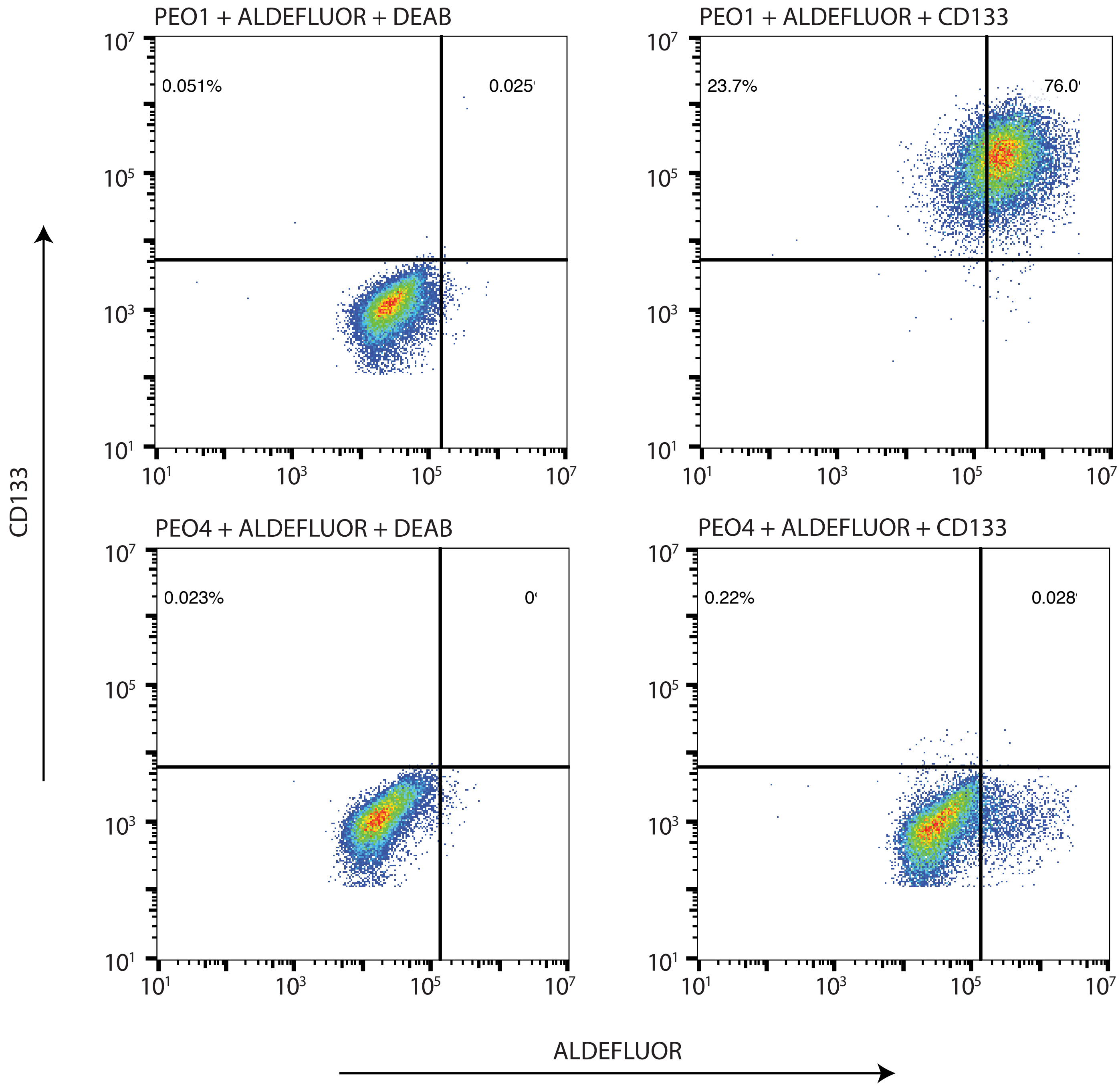

Supplement: Supplementary file 1 — Supplemental Materials and Methods [file 41417_2025_941_MOESM1_ESM.pdf]
